# A tiered strategy to identify relevant genetic variants in familial pulmonary fibrosis: a proof of concept for the clinical practice

**DOI:** 10.1038/s41431-024-01772-y

**Published:** 2025-01-02

**Authors:** Aitana Alonso-González, Ibrahim Véliz-Flores, Eva Tosco-Herrera, Silvia González-Barbuzano, Alejandro Mendoza-Alvarez, Helena Galván-Fernández, Leandro Sastre, Beatriz Fernández-Varas, Almudena Corrales, Luis A. Rubio-Rodríguez, David Jáspez, José M. Lorenzo-Salazar, Maria Molina-Molina, Felipe Rodríguez-de-Castro, Rafaela González-Montelongo, Carlos Flores

**Affiliations:** 1 Research Unit, Hospital Universitario Nuestra Señora de Candelaria, Instituto de Investigación Sanitaria de Canarias (IISC), Santa Cruz de Tenerife, Spain; 2 Universidad de Santiago de Compostela, Santiago de Compostela, Spain; 3 Instituto de Tecnologías Biomédicas, Universidad de La Laguna, Laguna, Spain; 4 Servicio de Neumología, Hospital Universitario de Gran Canaria “Dr Negrín”, Las Palmas de Gran Canaria, Spain; 5 Instituto de Investigaciones Biomédicas CSIC-UAM, Madrid, Spain; 6 Genomics Division, Instituto Tecnológico y de Energías Renovables, Santa Cruz de Tenerife, Spain; 7 Servei de Pneumologia, Laboratori de Pneumologia Experimental, IDIBELL, Barcelona, Spain; 8 Campus de Bellvitge, Universitat de Barcelona, Barcelona, Spain; 9 CIBER de Enfermedades Respiratorias (CIBERES), Instituto de Salud Carlos III, Madrid, Spain; 10 Facultad de Ciencias de la Salud, Universidad Fernando Pessoa Canarias, Las Palmas de Gran Canaria, Spain

**Keywords:** Genetic testing, Respiratory tract diseases

## Abstract

Idiopathic pulmonary fibrosis (IPF) is a progressive, late-onset disease marked by lung scarring and irreversible loss of lung function. Genetic factors significantly contribute to both familial and sporadic cases, yet there are scarce evidence-based studies highlighting the benefits of integrating genetics into the management of IPF patients. In this study, we performed whole-exome sequencing and telomere length (TL) measurements on IPF patients and their relatives. We then identified rare deleterious variants using three virtual gene panels encompassing IPF or TL genes with varying levels of evidence supporting their potential relationship with the disease. We identified 10 candidate variants in well-established disease genes, and these results were validated using two automatic prioritization tools (Exomiser and Franklin). Pathogenic variants were found in two telomere-related genes (*RTEL1* and *NAF1*), and both were associated with severe TL shortening. Our results suggest that this tiered virtual panel strategy is sufficiently robust and serves as a viable solution in clinical practice. It generates valuable genetic data which can be interpreted and validated with the expertise of a multidisciplinary team.

## Introduction

Idiopathic pulmonary fibrosis (IPF), the most common fibrotic interstitial lung disease (ILD), is a rare, chronic, late-onset disease, characterized by progressive scarring of the lung parenchyma and the irreversible loss of lung function [1]. The mechanisms that underlie IPF are incompletely understood, although an aberrant response of the alveolar epithelium to recurrent injury caused by triggering factors such as air pollution or other environmental exposures is supported [2].

Genetic factors play a significant role in both familial and sporadic forms, with many genetic studies providing valuable insight into disease etiology [3]. Rare variants in telomere maintenance-related genes, such as *TERT* and *TERC* [4]*, PARN, RTEL1* [5]*, NAF1* [6]*, DKC1* [7]*, TINF2* [8]*, ZCCHC8* [9], and *NOP10* [10], and the surfactant-related genes *SFTPA1/2* [11]*, SFTPC* [12], and *ABCA3* [13], are known to cause familial pulmonary fibrosis (FPF). Common variants in some of these genes have been also associated with risk in sporadic IPF in genome-wide association studies (GWAS) [14]. GWAS have also identified many other genetic risk loci, highlighting new genes related to immunity, the lung surfactant, cellular adhesion, and mechanotransductive functions [3, 14–16]. Therefore, these studies have established the presence of distinct molecular subtypes that exhibit varying trajectories concerning prognosis or response to treatment [17].

The implementation of precision medicine strategies in IPF patients is under development, while some approaches are currently being explored in clinical settings. One actionable diagnostic test involves telomere length (TL) measurement. This biomarker has proven to be a reliable predictor of survival and assists in stratifying patients for lung transplantation [18]. Another approach that may aid in achieving accurate diagnosis is genetic testing by DNA sequencing. In Spain this practice is not yet available as a standard for the clinical routine while in other countries it is primarily offered to patients with a family history suggestive of a telomerase dysfunction syndrome [19]. Given short TL is associated with germline variants in genes involved in telomere biology, both techniques are often performed in conjunction.

Here we devised a tiered strategy based on virtual gene panels including monogenic FPF-genes and TL-related genes and tested it using TL measurements and whole-exome sequencing (WES) data from 13 Spanish families affected by FPF to identify all relevant genetic variants underlying the disease pathogenesis.

## Materials and methods

### Study families

The study included family members residing in the Canary Islands (Spain) between 2020 and 2023 who met the following inclusion criteria: (1) a family history of ILD (i.e., with at least two family members having a confirmed diagnosis of IPF or other ILD); (2) at least one participant who has been diagnosed with FPF by a pulmonologist following the international clinical guidelines for ILD [20] available for genetic testing. During the clinical interview, demographics (age and sex) and clinical data (smoking, age at onset of the symptoms, and comorbidities) were collected from the affected family members. Peripheral blood samples for the WES analysis and buccal swab samples for the TL measures were also obtained from all participants.

The study received approval from the Ethics Committee of Hospital Universitario de Gran Canaria Dr. Negrín (2020-298-1) and was conducted in accordance with The Code of Ethics of the World Medical Association (Declaration of Helsinki). Written informed consent was obtained from all participants.

### TL measurement and analysis

Given the strong correlation between TL measures in blood cells, fibroblasts, and buccal cells [21, 22], DNA was isolated from mouth epithelial cells (oral swab) using a commercial DNA isolation kit (Isohelix, Cell Projects Ltd.). TL relative measures were obtained from all participants (whenever possible) at the Instituto de Investigaciones Biomédicas (CSIC/UAM) using quantitative polymerase chain reaction (qPCR) as described elsewhere [23].

Because TL varies with age, Z-score values were calculated to enable comparisons of TLs across individuals of different ages. The Z-score compared the Telomere Shortening (T/S) ratio value of each individual to the age-matched mean and standard deviation (SD) of control values. Severe TL reduction was denoted when Z-score was below the 10th percentile.

### Exome sequencing, variant annotation and filtering

Libraries were prepared from peripheral blood genomic DNA using the Illumina DNA Prep with Enrichment kit (Illumina Inc., San Diego, CA) following the methods described elsewhere [24], and sequencing was performed on a Illumina HiSeq 4000 or Illumina NovaSeq 6000 sequencing systems (Illumina Inc.) using 75 bp or 100 paired end reads, respectively.

The detection of small insertions/deletions (<50 bp) and single nucleotide variants (SNVs) was carried out with an in-house bioinformatics pipeline based on GATK HaplotypeCaller v3.8 (https://gatk.broadinstitute.org/hc/en-us/articles/360037225632-HaplotypeCaller) using the GRCh37/hg19 reference version of the human genome. Variants were annotated using ANNOVAR v18.04.16 (https://annovar.openbioinformatics.org/en/latest/) and then filtered based on population frequency and their predicted impact at the protein level. Additional details can be found in the **Supplementary material**.

### Virtual gene panels

Genes were included after a rigorous review of published evidence in the literature and online databases.

1. Diagnostic gene panel for FPF (Panel A).
  A virtual gene panel tailoring monogenic forms of FPF was designed following recommendations from the American College of Medical Genetics and Genomics (ACMG) [25]. Genes were considered for inclusion if rare deleterious genetic variants were found in a substantial proportion of FPF cases. Additionally, genes that emerged as relevant to IPF through large-scale genetic studies, such as *KIF15* [26] or *SPDL1* [27], were also incorporated. This resulted in a concise list of 14 genes which were categorized into two broad categories: telomere-related genes and non-telomere related genes (Table 1).

**Table 1:** Genes related to familial forms of IPF included in Panel A.

Gene	Inheritance	Phenotype (OMIM)			Category	Gene-disease evidence	References
*TERC*	AD	Dyskeratosis congenita, autosomal dominant 1; Pulmonary fibrosis and/or bone marrow failure syndrome, telomere-related, 1			Telomere-related	Strong	[41]
*TERT*	AD, AR	Dyskeratosis congenita, autosomal dominant 2; Pulmonary fibrosis and/or bone marrow failure syndrome, telomere-related, 1			Telomere-related	Definite	[41]
*TINF2*	AD	Dyskeratosis congenita, autosomal dominant 2; Revesz syndrome			Telomere-related	Moderate	[8]
*DKC1*	XLR	Dyskeratosis congenita, X-linked			Telomere-related	Definite	[7]
*RTEL1*	AD, AR	Dyskeratosis congenita, autosomal dominant 4, autosomal recessive 5; Pulmonary fibrosis and/or bone marrow failure syndrome, telomere-related, 3			Telomere-related	Strong	[5]
*PARN*	AD	Dyskeratosis congenita autosomal recessive 6; Pulmonary fibrosis and/or bone marrow failure syndrome, telomere-related, 4			Telomere-related	Moderate	[5]
*NAF1*	AD	Pulmonary fibrosis and/or bone marrow failure syndrome, telomere-related, 7			Telomere-related	Strong	[6]
*ZCCHC8*	AD	Pulmonary fibrosis and/or bone marrow failure syndrome, telomere-related, 5			Telomere-related	Moderate	[9]
*SFTPC*	AD	Surfactant metabolism dysfunction, pulmonary, 2			Non-telomere related	Strong	[12]
*SFTPA2*	AD	Interstitial lung disease 2			Non-telomere related	Strong	[11]
*ABCA3*	AR	Surfactant metabolism dysfunction, pulmonary, 3			Non-telomere related	Strong	[13]
*SFTPA1*	AD, AR	Interstitial lung disease 1			Non-telomere related	Strong	[42]
*SPDL1*	AD	IPF			Non-telomere related	Limited	[27]
*KIF15*	AD	IPF			Non-telomere related	Limited	[26]

Evidence levels to describe the strength of evidence supporting a gene-disease association were defined following criteria from the ClinGen Gene Curation Working Group. We only included genes whose supporting evidence was Definite, Strong, or Moderate, except for *SDPL1* and *KIF15* for which there is still limited evidence to support its role in the disease.

AD autosomal dominant, AR autosomal recessive, XLR X-linked recessive, IPF idiopathic pulmonary fibrosis, OMIM Online Mendelian Inheritance in Man.
2. Gene panel associated with ILD (Panel B).
  An extended virtual gene panel was designed, covering other ILD genes, genes associated with dyskeratosis congenita, and genes associated with syndromes such as Hermansky-Pudlak syndrome or Tuberous sclerosis which may also present with PF. Moreover, genes identified as associated with IPF in GWAS studies were also added. This includes *MUC5B* since its promoter polymorphism (rs3570950-T) is the strongest common genetic risk factor known for IPF (Supplementary Table 1).
3. Gene panel associated with TL (Panel C).

Given the well-known relationship between IPF and TL, a specific virtual gene panel including genes related to telomere maintenance was designed. It included genes absent from panel A or B, including others linked to TL biology identified through GWAS studies or that were annotated with the telomere maintenance ontology term (GO:0000723) (Supplementary Table 1).

### Tiered approach for data analysis

**Fig. 1:**
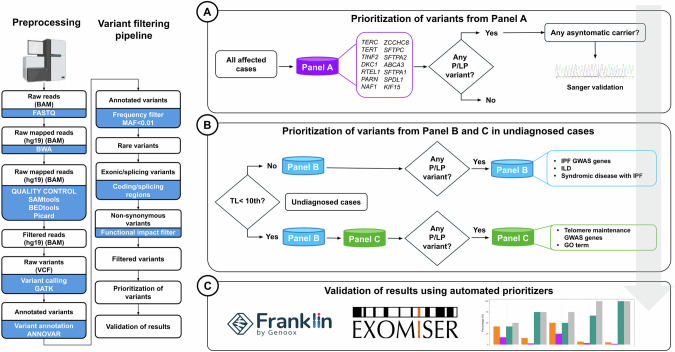
Workflow of data analysis. Exonic, non-synonymous rare variants (AF < 0.01) were analyzed following a tiered approach. Virtual gene panels (Panel **A, B**, and **C**) were applied sequentially to identify candidate variants in genes associated with interstitial lung disease or genes related to telomere maintenance function. Two automated prioritization tools (Exomiser and Franklin) were applied to validate the findings of using the virtual gene Panel **A**.

A tiered approach was adopted for the analysis of the filtered variants. The algorithm is summarized in Fig. 1.

To reduce the number of variants for prioritization, virtual gene panels were applied and resulting variants were classified according to the variant interpretation guidelines outlined by the ACMG [28]. Briefly, all families were interrogated for variants in genes from Panel A. If no P or LP variants were identified in affected members, then the analysis was extended to assess variants from Panel B genes. The analysis of variants in Panel C genes was restricted to those affected cases with severe telomere shortening (<10th percentile) in which no P/LP variants were found using Panel A or B.

Additionally, two of the best-performing public automated variant prioritization tools, Exomiser v13.0.0 (https://github.com/exomiser/Exomiser) and Franklin (https://franklin.genoox.com/clinical-db/home), were used to evaluate if they ranked the expected relevant variants for each patient on top of the variant list (top first or top five) when searching across whole exome data. Additional details are available in **Supplementary material**.

### Sanger validation

Variants classified as VUS, LP, or P with plausible clinical relevance, underwent validation through direct Sanger sequencing (BigDye Terminator v3.1 cycle sequencing kit; Thermo Fisher Scientific) of PCR amplicons. This assessment was performed using the Macrogen (Spain) sequencing services based on custom amplicon designs (Supplementary Table 2).

### Statistical analysis

Descriptive statistics were provided as mean (SD) or median (interquartile range) and valid percentage for continuous and categorical data, respectively. The relationship between TL and heterozygous status was assessed using a Fisher’s exact test, where only the P/LP/VUS-LP were considered for the comparison. To compare TL means (expressed as percentiles) among groups, the Welch t-test was used. All statistical analyses were performed using R statistical analysis software, version 4.3.1.

## Results

### Study sample and sequencing analysis

The study sample consisted of 61 individuals of European ancestry from 13 families residing in the Canary Islands (Spain) (Supplementary Fig. 1). In all of them, an inheritance pattern of FPF consistent with autosomal dominant transmission was observed. Out of these, 16 participants (8 males and 8 females) received a confirmed diagnosis of FPF and were considered as affected members. Notably, three families (families 5, 7, and 12) had two affected among its members. The affected patients were aged between 46 and 85 years old (mean 63.9 ± 12). At the moment of the clinical evaluation, 45 participants were considered unaffected, although they are currently monitored by a pulmonologist in the clinical consultation.

WES yielded an average of 10.1 gigabases (Gb) per participant, with 87.9% of on-target regions covered at >20X depth. Detailed coverage for Panel A genes can be found in the Supplementary Table 3.

### Impact of using a tiered approach for capturing relevant genetic variants in FPF

The aim of this approach was to capture all relevant genetic variants underlying FPF from the WES results. In each family, candidate variants were prioritized in affected members and their presence was subsequently tested in apparently unaffected relatives to reveal asymptomatic heterozygotes. B, LB, and VUS-LB were excluded from further analyses.

Initially, the 16 affected members from the 13 families were assessed for coding, non-synonymous rare variants (AF < 0.01) from Panel A genes. The average number of variants (± SD) per affected case retained for interpretation was 1.44 (± 1.31) (Range: 0–5) demonstrating the effective approach for filtering and prioritization (Fig. 1).

**Fig. 2:**
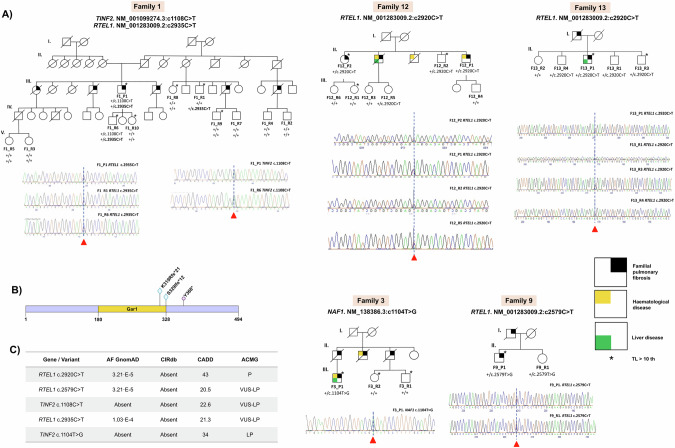
Pedigrees of the families carrying relevant genetic variants in telomere-related genes. **A** *TINF2* and *RTEL1* genotypes are shown below the sequenced individuals from family 1. Sequence electropherograms (bottom) supporting the variants *TINF2* c.1108 C > T and *RTEL1* c.2935 C > T are shown. *RTEL1* genotypes are shown below the sequenced individuals from family 12. Sequence electropherograms (bottom) supporting the variant c.2920 C > T are shown. *RTEL1* genotypes are shown below the sequenced individuals from family 13. Sequence electropherograms (bottom) supporting the variant c.2920 C > T are shown. *NAF1* genotypes are shown below the sequenced individuals from family 3. Sequence electropherogram (bottom) supporting the variant c.1104 T > G is shown. *RTEL1* genotypes are shown below the sequenced individuals from family 9. Sequence electropherogram (bottom) supporting variant c.2579 C > T are shown. **B** Schematic representation of NAF1 protein (NP_612395.2) depicting the Gar1 conserved domain (as defined by the NCBI conserved domain database) and the two variants previously found to cause IPF, and emphysema described by Stanley et al. [14]. (K319Rfs*21; S329Ifs*12) and the variant found in F3_P3 in this study (Y368*). A ‘+´ symbol on the genotypes stands for the reference allele of all families. **C** Population frequency data, in silico predictor CADD score, and ACMG classification of candidate variants.

In total, 10 variants were found to predict effects in well-established disease genes (Table 2). Eight of them were identified in telomere-related genes and six variants affected *RTEL1*. Three variants were classified as LP/P. The P variant *RTEL1* c.2920 C > T was identified in affected patients from families 12 and 13 (Table 2, Fig. 2A). The variant was also present in two asymptomatic relatives from family 12, and three members from family 13 aged 25 to 59 years old. The novel LP variant *NAF1* c.1104 T > G was identified only in the affected member from family 3 (Table 2, Fig. 2A, Fig. 2B). Additionally, variants found in families 1 and 9 were classified as VUS-LP (Table 2, Fig. 2A, Fig. 2C). The remaining variants were considered of unknown significance, and they were found in five patients from four families (Table 2, Supplementary Fig. 2). Additional details on these variants can be found in **Supplementary material**. Clinical characteristics of affected individuals carrying variants in genes from Panel A are summarized in Table 3.

**Table 2:** Pathogenic (P), likely pathogenic (LP), and variants of uncertain significance (VUS) identified in the families with FPF members through the tiered approach.

Case ID	Family ID	TL (Percentile)	Gene	HGVS	Amino acid change	Functional effect	Zygosity	ACMG class
F8_P1	8	25-50	*RTEL1*	NM_001283009.2:c.3470 C > A	p.Pro1157His	Missense	Het	VUS
F12_P1 F12_P2	12	10-25 <1	*RTEL1*	NM_001283009.2:c.2920 C > T	p.Arg974*	Nonsense	Het	P
F13_P1	13	<10	*RTEL1*	NM_001283009.2:c.2920 C > T	p.Arg974*	Nonsense	Het	P
F9_P1	9	<10	*RTEL1*	NM_001283009.2:c.2579 C > T	p.Ser860Phe	Missense	Het	VUS-LP
F11_P1	11	10-25	*SFTPA2*	NM_001098668.4:c.482 G > A	p.Arg161His	Missense	Het	VUS
F1_P1	1	<1	*TINF2*	NM_001099274.3:c.1108 C > T	p.Pro370Ser	Missense	Het	VUS-LP
F1_P1	1	<1	*RTEL1*	NM_001283009.2:c.2935 C > T	p.Arg979Trp	Missense	Het	VUS-LP
F3_P1	3	<1	*NAF1*	NM_138386.3:c.1104 T > G	p.Tyr368*	Nonsense	Het	LP
F6_P1	6	50-75	*SPDL1*	NM_017785.5:c.892-2 A > G	.	Splicing	Het	VUS
F7_P1 F7_P2	7	NA 10-25	*TERT*	NM_198253.3:c.2885 G > A	p.Arg962His	Missense	Het	VUS

*VUS* variants of uncertain significance, *P* pathogenic, *VUS-LP* variants of uncertain significance, likely pathogenic, *LP* likely pathogenic, *TL* telomere length, *HGVS* Human Genome Variation Society, *Het* heterozygous, *ACMG* American College of Medical Genetics.

In the first tier, rare deleterious variants were identified in index cases using a virtual gene panel (Panel A).

**Table 3:** Clinical characterization and relative telomere length for individuals with variants identified in genes from Panel A.

ID patient	F12_P1	F12_P2	F13_P1	F3_P1	F1_P1	F9_P1	F11_P1	F6_P1	F8_P1	F7_P1	F7_P2
Gender	Male	Female	Male	Male	Male	Male	Female	Male	Female	Male	Female
Age (years)	56	64	56	46	83	67	64	74	67	46	58
Smoking	Yes	No	Yes	No	Yes	No	No	Yes	No	No	Yes
Diagnosis	IPF	IPF	Emphysema/IPF	IPF	Emphysema/IPF	IPAF	IPF	IPF	IPF	Unclassifiable PF	Emphysema/IPF
Disease onset (years)	55	61	50	44	79	65	61	72	66	34	57
Family history	Two brothers with fibrotic ILD, hepatic disorder and bone marrow failure	Two brothers with fibrotic ILD, hepatic disorder and bone marrow failure	Brother with ILD and asthma. Father with unclassifiable PF	Father and two uncles with ILD	Three brothers and two cousins with ILD	Father with fibrotic ILD	Aunt and cousin with fibrotic ILD	Brother with ILD	Mother with fibrotic ILD	Mother with fibrotic ILD and sister with ILD	Mother and brother with ILD
Lung transplant	Candidate	Candidate	Candidate	Candidate	No candidate	Candidate	Candidate	No candidate	Candidate	No candidate	Candidate
Initial symptoms	Dyspnea	Dyspnea	Dyspnea	Dyspnea and cough	Dyspnea and cough	Cough	Dyspnea and cough	Dyspnea and cough	Dyspnea and cough	Dyspnea	Dyspnea and cough
Time to death from diagnosis (months)	24	–	–	48	60	–	–	–	–	12	–
Comorbidities	Thrombocytopenia	Asthma	Cryptogenic hepatic cirrhosis, type 2 diabetes	Epilepsy, cryptogenic liver disease, Thrombocytopenia, coxarthrosis	Treated tuberculosis; Lumbar canal stenosis L4-L5	Hypertension, Avascular necrosis, polyarthritis, dyslipedemia	Gastroesophageal reflux	Hypertension, type 2 diabetes, dyslipidemia	Hypertension, dyslipidemia, chronic autoimmune thyroiditis	Cryptogenic cirrhosis	Dyslipidemia/ Migraine
Treatment	Oxygen/ Antifibrotics Corticoesteroids	Antifibrotics	Antifibrotics	Antifibrotics	Oxygen/ Antifibrotics	Corticosteroids/ Antifibrotics	Antifibrotics	Antifibrotics/ Oxygen	Corticosteroids		
HRCT	UIP pattern	UIP pattern	Emphysema/ UIP pattern	UIP pattern	Emphysema/UIP pattern	UIP pattern	UIP pattern	UIP pattern	UIP pattern	Inconsistent with UIP	UIP pattern
FVC (%)	48	78	45	68	63	66	87	69	71	81	86
FEV1 (%)	49	79	54	70	70	73	94	68	72	57	84
DLCO (%)	42	60	48	36	36	58	62	49	46	12	76
Telomere length	10-25	<1	<10	<1	<1	<10	10-25	50-75	<1	unknown	10-25
Variant	*RTEL1* c.2920 C > T	*RTEL1* c.2920 C > T	*RTEL1* c.2920 C > T	*NAF1* c.1104 T > G	*TINF2* c.1108 C > T *RTEL1* c.2935 C > T	*RTEL1* c.2579 C > T	*SFTPA2* c.482 G > A	*SPDL1* c.892-2 A > G	*RTEL1* c.3470 C > A	*TERT* c.2885 G > A	*TERT* c.2885 G > A

*IPF* idiopathic pulmonary fibrosis, *ILD* interstitial lung disease, *UIP* usual interstitial pneumonia, *HRCT* high-resolution computed tomography, *FVC* forced vital capacity, *DLCO* diffusing capacity of the lung for carbon monoxide, *IPAF* interstitial pneumonia with autoimmune features.

As P or LP variants were identified using Panel A in families 3, 12, and 13, they were not considered for further analysis. In the remaining 10 families, pathogenicity of rare potential deleterious variants from Panel B genes was assessed (Fig. 1). For this tier, the average number of filtered variants was 6 (± 3.63) (Range: 0–12). Panel C was only used in those families with affected cases exhibiting severe TL reduction and no P/LP variants identified in previous steps (i.e., in patients F1_P1 and F9_P1) (Fig. 1).

A total of 18 variants classified as VUS were prioritized in eight individuals (Supplementary Table 4) and they were affecting 13 genes (11 from Panel B and two from Panel C). Eight variants were found in IPF genes which has been reported in existing GWAS studies (*MUC5B, ACTRT3, AKAP13, MAD1L1*, and *RAPGEF2*). Other seven variants were identified in genes associated with syndromic diseases which may also develop ILD (*HPS3, WRAP53, GLA, DPP9, FAM111B, TSC1*, and *LIG4*). However, they were not consistent with the phenotype of the affected patients. Two variants were found in *SFTPD*, which has not been associated before with ILD despite the encoded protein participates in the surfactant metabolism.

**Fig. 3:**
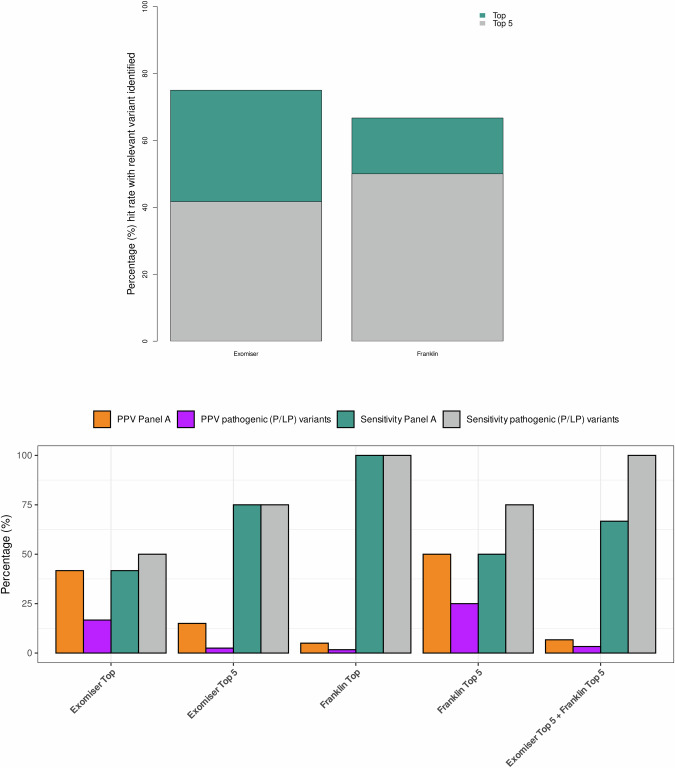
Performance of automated phenotype-driven approaches in FPF. **A** Cumulative percentage of correctly prioritized variants from Panel **A** considering only the top-first and top-five ranked variants. **B** Positive predictive values (PPV) and sensitivity of Exomiser and Franklin prioritizers when used individually and combined. PPV and sensitivity were calculated considering all candidate variants or only pathogenic (P/LP) variants within genes from Panel **A**.

Taken together, all relevant variants of the 13 families were identified in genes included in a panel tailoring monogenic IPF or FPF which included 14 genes in total. No other relevant variant was found using the extended panels (B or C). These results were validated using automated-prioritization tools Exomiser and Franklin (see **Supplementary material** and Supplementary Table 5) which prioritized eight and seven of the manually prioritized variants in genes from Panel A among their top five priority ranks, respectively (Fig. 3). None of the variants that were manually prioritized from panels B or C was prioritized by Exomiser or Franklin, except those for *SFTPD* which were prioritized by Exomiser in both heterozygotes.

### Utility of TL for capturing relevant genetic variants in IPF families

**Fig. 4:**
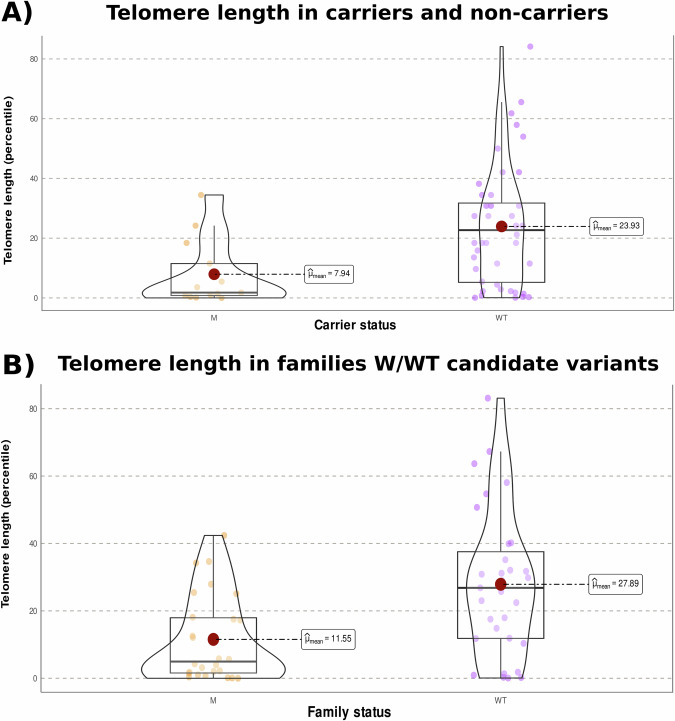
Violin plots showing the distribution of telomere length (TL). **A** TL percentile means among heterozygotes for variants in telomere-related genes (M) and non- heterozygotes (WT) irrespective of the affected status. **B** TL percentile means among subjects (patients and unaffected) from families where a telomere-related variant was found (M) and among subjects from families without variants in telomere-related genes (WT). The mean percentile of TL per group is indicated.

TL assessment was conducted in 16 patients and 42 asymptomatic relatives (Supplementary Fig. 1). Severe TL shortening was denoted in five patients from five families. The proportion of relevant genetic variants from Panel A was significantly lower (Fisher’s exact test, *p* = 0.002) in patients with TL > 10th percentile (1/6) than in patients with severe TL shortening (5/6). Three of these were heterozygotes for P/LP variants in *RTEL1* and *NAF1* genes while the other two were heterozygotes for VUS-LP in *RTEL1* and *TINF2* genes (Fig. 2A). When considering all individuals (irrespective of the affected status) the proportion of relevant genetic variants was significantly higher (Fisher’s exact test, *p* = 0.02) in individuals with severe TL shortening (8/21) compared to individuals with TL > 10th percentile (5/36) and the mean of TL among heterozygotes was in the 7.94 percentile (Fig. 4A). We also found that the mean TL percentile was significantly lower (Welch *t*-test, *p* = 9.0 × 10^−^^4^) among patients and unaffected from families with a telomere-related variant (11.55) than in subjects from families without a telomere-related variant (27.89). This finding is consistent with the known inheritance of short telomeres in offspring of telomerase mutation heterozygotes (Fig. 4B).

### Genotype-phenotype correlations in patients carrying P/LP variants and their impact on relatives

In the three families where a P or LP variant in a telomere-related gene was identified (F12, F13, and F3) there was a prior suspicion of a telomere syndrome based on the documented phenotypes of some family members. Overall, FPF was the most common phenotype while the expression of extra-pulmonary symptoms (such as hematological and liver disease) and TL varied across affected patients and unaffected carriers (Table 3, Fig. 2A).

The well-known variant (c.2920 C > T; p.Arg974*) (**Supplementary methods**) in *RTEL1* was identified in affected members from families 12 and 13 (F12_P1, F12_P2, and F13_P1) and it was also present in two asymptomatic relatives from family 12 (F12_R2 and F12_R5), and three from family 13 (F13_R1, F13_R3 and F13_R4) aged 25 to 59 years old (Fig. 2A).

In family 12, F12_P1 presented thrombocytopenia along with fibrotic ILD. In contrast, F12_P2 did not exhibit any extra-pulmonary symptoms at the time of evaluation although she showed severe TL shortening. Family history was available for two brothers from both affected patients. One brother was diagnosed with fibrotic ILD, hepatic disorder, and severe thrombocytopenia, while the other died of bone marrow failure (Table 3, Fig. 2A). In family 13, F13_P1 underwent liver transplantation due to cryptogenic hepatic cirrhosis before being diagnosed with FPF. In asymptomatic relatives, the pathogenic variant co-segregated with severe TL shortening only in F12_R2 and F13_R3. Interestingly, four relatives were non-carriers yet one of them exhibited severe TL reduction (Table 3, Fig. 2A).

The novel LP variant in exon 8 of *NAF1* (c.1104 T > G; p.Tyr368Ter) was identified in affected patient F3_P1 and was absent in unaffected relatives (F3_R1 and F3_R2) although they also had severe TL shortening (Fig. 2A).

In F3_P1, hematological and hepatic alterations preceded IPF, the first presenting before the age of 30 years, and receiving the diagnosis of IPF at 44 years of age. His father and two uncles (not participating in this study) were also affected by FPF. For these relatives, the onset of PF occurred at a mean age of 61 years and the mean survival from the date of diagnosis was 15 months. Multisystem involvement was rare, except for one of the uncles, which also presented with megaloblastic anemia, and the survival time was shorter (Table 3, Fig. 2A).

### Performance of the tiered strategy for identifying relevant variants in 13 FPF families

Our strategy identified four FPF patients and five asymptomatic heterozygotes who carried a P or LP variant in telomere-related genes included in Panel A. Considering families as the unit, P or LP variants were identified in three of them, providing a conservative molecular diagnostic yield of finding relevant genetic variants of 23.1% (95% Confidence Interval [CI]: 8.2–50.0). Additional relevant variants, which were classified as VUS-LP, were identified in two other families. Adding these two families to the estimate increased the molecular diagnostic of finding relevant genetic variants to 38.5% (95% CI: 18.0–64.0).

## Discussion

In this study, we developed a tiered strategy by constructing virtual gene panels that encompass FPF or TL genes with varying level of evidence supporting their relationship with the disease [25]. We assessed its performance for identifying relevant genetic variants of FPF in subjects residing in the Canary Islands with WES and TL data and found that, if implemented in the daily practice, it provides a valuable tool for the aid in the genetic characterization of patients. Interpreting these data in the context of a multidisciplinary team with expertise in clinical genetics and ILD could have a significant impact on patients and relatives.

Targeted sequencing of a limited set of genes enables the identification of relevant variants with high sensitivity while reducing the cost and time of diagnostic testing [29]. However, its benefits are limited when the genetic basis of a condition is not yet well defined, and new genes are continuously being discovered through ongoing genetic research, as is the case of ILD. As the cost of sequencing decreases, whole-exome and whole-genome sequencing is replacing the use of targeted gene panels in healthcare settings, allowing for the interrogation of the vast majority of known protein coding human genes [30]. The major challenge is then related with the workload of interpretation of findings due to the incidental findings and the high burden of VUS. Here we show that the design of virtual gene panels on top of whole-exome information offers benefits. They preserve diagnostic yield while reducing interpretative variant workload [31] and can be easily updated as new evidence supports gene-disease associations [32].

Our first tier consisted of a concise gene panel including those genes with a definite, strong, and moderate level of evidence. Additionally, we considered *KIF15* and *SPDL1* as they were recently supported to be IPF genes [26, 33]. Relying on this first-tier gene panel, the average number of variants selected for prioritization was just one per family and we successfully identified the most likely relevant variants among them, reaching a conservative diagnostic yield of 23.1% in our cohort. These results were comparable with those obtained using two of the best-performing public phenotype-driven automated approaches considering the whole exome (https://github.com/genomicsITER/benchmark-germline-variants-prioritizers), supporting that the tiered virtual panel strategy was sufficiently robust.

Our results support that Panel A is currently appropriate for capturing relevant genetic variants in FPF. While doing so, we exposed technical considerations of WES enrichment kits which could be taken into account when implementing a similar approach [25]. For example, the fraction of bases covered at >10X depth was 55.2% in *ZCCHC8*, while it was 90.4% for *KIF15*. In addition, *TERT*, one of the genes accumulating most of the relevant variants in FPF in the literature, reached a relatively low depth of coverage in exonic regions with only 63.1% of the bases being covered at >10X (Supplementary Table 3). Thus, the choice of the commercial whole-exome enrichment capture solution to implement the described procedure should be carefully considered when designing virtual gene panels [24]. Our comparisons of three commercial enrichment solutions revealed that Illumina DNA Prep with Exome 2.5 Enrichment showed the optimal results when considering fraction of bases covered at >10X. In addition, caution should be taken when using some of the enrichment solutions since some do not allow to recover sequencing data from the *TERC* gene (Supplementary Fig. 3).

We learned several indications for using TL measures as a companion for diagnostics in patients with FPF, as it provides distinct and somehow complementary information from DNA sequencing. Severe TL shortening was considered as a supporting pathogenic criterion based on all the collected evidence for the families. For instance, severe TL reduction in heterozygotes for variant *NAF1* c.1104 T > G supports a damaging effect. Because of that, we were able to reveal the third deleterious variant related to FPF that has been described to date in the *NAF1* gene [6]. In addition, we observed that it was significantly more likely to identify a relevant variant in a telomere-related gene in affected individuals with severe TL shortening, as it has been widely described [34–36]. Future studies with larger sample sizes should clarify whether TL measures might be advantageous for selecting individuals who should undergo gene sequencing.

We sequenced not only affected members but also first-degree relatives. This allowed to identify six otherwise asymptomatic heterozygotes for a widely reported pathogenic variant in *RTEL1* (c.2920 C > T; p.Arg974*). Given the variable penetrance of the disease and its expressivity, offering genetic testing to first-degree relatives of affected FPF patients remains a topic of ongoing debate. However, relatives of patients with sporadic and FPF have high rates of ILD and interstitial lung abnormalities (ILA) [37], and disease progression is common among patients with ILA [38]. Although further studies are needed, genetic testing could assist in identifying high-risk relatives who may benefit from imaging studies such as chest computed tomography which may identify early signs of ILA. Besides, genetic testing interventions may influence lifestyle changes (i.e., reduce smoking exposure) and preliminary results suggest that initiating an antifibrotic therapy in early stages of the disease offers better results [39].

In this study, a significant limitation arises from the absence of sequencing data for multiple affected cases within each of the available families, making it especially challenging to determine the pathogenicity of variants. Consequently, we were unable to test the co-segregation of candidate variants with the disease. This, combined with the lack of functional data, explains why most of the rare variants identified in this study were considered of unknown significance. Furthermore, the late onset and incomplete penetrance of the disease make it a poor election to consider asymptomatic relatives as suitable controls. In the case of families considered for this study, the family members are still under close follow-up to be able to detect the potential emergence of new affected members. Thus, we expect that a more accurate classification of VUS will be obtained in the future, as clinical information from other family members is provided.

Among the strengths, we highlight the focus on families from an isolated population, with a unique genetic background in Europe resulting from the historical admixture of North Africans, Europeans, and Sub-Saharan African populations [40]. This unique genetic background may contribute to the higher prevalence of some rare diseases in this archipelago [40]. Although we were not able to identify new genetic causes of FPF, we found two otherwise unrelated families sharing the same P variant. This variant does not represent a founder monogenic variant since it has been described elsewhere in other populations. However, its prevalence might be increased in the Canary Islanders. This fact encourages to continue recruiting and genetically testing incident FPF cases in the population.

In conclusion, we have devised a tiered strategy based of virtual gene panels for identifying relevant genetic variants in affected FPF families. This allowed us to successfully detect rare deleterious variants in genes previously linked to FPF, demonstrating that our approach is sufficiently robust. The analysis of additional families will be needed to refine and update this strategy before considering it a valuable solution for implementing genetic testing into the clinical practice.

## Supplementary information

Supplementary material

## Data Availability

Sequence data generated during this study have been deposited at the European Genome-phenome Archive (EGA), which is hosted by the EBI and the CRG, under accession number EGAD50000001152. All variants of clinical interest identified during the current study are available in tables and supplemental tables.
