# Supplementary material for: A tiered strategy to identify relevant genetic variants in familial pulmonary fibrosis: a proof of concept for the clinical practice
Source: Eur J Hum Genet. 2025 Jan 2;33(11):1509–19. doi: 10.1038/s41431-024-01772-y (PMC12583455; doi:10.1038/s41431-024-01772-y)
Supplement: Supplementary file 1 — Supplementary material [file 41431_2024_1772_MOESM1_ESM.docx]

**Supplementary material**

**A tiered strategy to identify relevant genetic variants in familial pulmonary fibrosis: a proof of concept for the clinical practice**

**Supplementary Methods**

1. **Detailed description of the bioinformatics pipeline**
2. **Variant filtering algorithm**

**Supplementary Results**

1. **Classification of genetic variants identified in genes from Panel A.**
2. **Coverage of genes from Panel A in three commercial whole-exome enrichment solutions.**
3. **Validation of results in FPF using automated bioinformatic prioritization tools.**

**Supplementary Tables**

**Supplementary Table 1.** Genes related to ILD, IPF susceptibility genes and telomere maintenance included in Panels B and C.

**Supplementary Table 2.** Sanger sequencing details of gene regions containing the prioritized variants from Panel A and B (*SFTPD*).

**Supplementary Table 3.** Fraction of bases (%) covered at 10X depth in FPF patients from this study.

**Supplementary Table 4.** List of VUS identified in the study using virtual Panels B and C.

**Supplementary Table 5.** Human Phenotype Ontology (HPO) terms used for recording phenotypes associated with IPF in Exomiser and Franklin.

**Supplementary Figures**

**Supplementary Figure 1.** Pedigrees from the 13 families included in the study.

**Supplementary Figure 2.** Pedigrees of the families carrying variants of uncertain significance in genes from Panel A.

**Supplementary Figure 3.** Proportion of bases covered on average per gene (Panel A).

**Supplementary References**

**Supplementary methods**

1. **Detailed description of the bioinformatics pipeline**

Genomic DNA was purified from peripheral blood using a commercial column-based DNA extraction kit (GE Healthcare, Chicago, IL). Sample concentrations were measured on the Qubit 3.0 fluorometer using the Qubit dsDNA HS Assay with the corresponding dsDNA HS Assay kit (Thermo Fisher Scientific, Waltham, MA). Libraries were prepared using the Illumina DNA Prep with Enrichment kit (Illumina Inc., San Diego, CA) following the methods described elsewhere (1). Library sizes were checked on a TapeStation 4200 (Agilent Technologies, Santa Clara, CA) and their concentration determined by the Qubit dsDNA HS Assay (Thermo Fisher). Sequencing of libraries was conducted along with 1% of a PhiX control V3 (Illumina Inc.) to an average depth >100X on Illumina HiSeq 4000 or Illumina NovaSeq 6000 sequencing systems (Illumina Inc.) using 75 bp or 100 paired end reads, respectively, at Instituto Tecnológico y de Energías Renovables (ITER, Santa Cruz de Tenerife, Spain).

Sequence reads were preprocessed as detailed elsewhere (1). Briefly, we used bcl2fastq v2.18 to perform sample demultiplexing and BWA-MEM v0.7.15 (<https://github.com/lh3/bwa>) to align reads to GRCh37/hg19 reference. Resulting BAM files were assessed with SAMtools v1.3 (<http://www.htslib.org>) and Picard v2.10.10 (<https://broadinstitute.github.io/picard/>) for quality control steps. Small insertions/deletions (<50 bp) and single nucleotide variants (SNVs) were identified using an in-house bioinformatics pipeline based on GATK HaplotypeCaller v3.8 (<https://gatk.broadinstitute.org/hc/en-us/articles/360037225632-HaplotypeCaller>). This pipeline follows the Best Practices recommendations for germline variant calling and its description is publicly available (<https://github.com/genomicsITER/benchmarking/tree/master/WES>). Detected variants were annotated for genetic RefSeq based annotation, functional consequences, population frequency, links with disease based on ClinVar and The Human Gene Mutation Database, and several pathogenicity scores including the Combined Annotation-Dependent Depletion (CADD) using ANNOVAR v18.04.16 (<https://annovar.openbioinformatics.org/en/latest/>). Analyses were conducted in the TeideHPC Supercomputing facility (<http://teidehpc.iter.es/en>).

1. **Variant filtering algorithm**

To prioritize variants, the following algorithm was applied. First, a threshold for population allele frequency (AF) was set at 0.01 and variants exceeding that value were filtered out. For that purpose two databases were used: an internal variant frequency database obtained from the whole exomes of 920 unrelated population controls from the Canary Islands (CIRdb) (2), and gnomAD v2.1.1 (<https://gnomad.broadinstitute.org/>) (N = 125,748). Then, variants were filtered based on their predicted impact at the protein level. Specifically, missense, nonsense, frameshift (insertion and deletion), non-frameshift (insertion and deletion), stop-loss, start-loss and splice site disrupting variants were retained. Variants located in intronic regions or within the 5’ or 3’ untranslated regions (UTRs) were excluded for further consideration except those synonymous and intronic variants with potentially harmful effect on splicing. The latter were analyzed using SQUIRLs v1.3.0 software, prioritizing them by the internal pathogenic score (<https://squirls.readthedocs.io/en/master/running.html>). Non-coding RNA *TERC* gene variants were also considered for prioritization if they satisfied an AF<0.01.

**Supplementary Results**

1. **Classification of genetic variants identified in genes from Panel A**

The well-known variant (c.2920C>T; p.Arg974*) in *RTEL1* was identified in affected members from families 12 and 13 (F12_P1, F12_P2 and F13_P1) (**Table 2**, **Figure 2A**). This variant is predicted to cause a truncated or absent protein product as evidenced by functional assays and has been widely reported in patients diagnosed with Dyskeratosis congenita, familial interstitial pneumonia and/or IPF (ClinVar variation ID:42020). For all the above, and the documented phenotypes described in both families **(Table 3, Figure 2A, 2C),** the variant was classified as P.

The novel LP variant in exon 8 of *NAF1* (c.1104T>G; p.Tyr368Ter) identified in F3_P1 from family 3 predicts the presence of a premature stop codon (**Table 2**, **Figure 2A, 2B**). This variant is absent from public population databases (gnomAD; n=125,748), variant annotation search engines (TOPMed, Ensembl Variant Effect Predictor, and VarSome), and local population controls (CIRdb, n=920). The CADD predictor also supported high impact (CADD = 32; MSC_CADD score = 8.93) (**Figure 2C**). The clinical characteristics described in the family also supported its pathogenicity (**Table 3**, **Figure 2A**).

Missense heterozygous variants in telomere-related genes were found in the remaining two affected individuals with severe TL shortening. The variants *TINF2* c.1108C>T and *RTEL1* c.2935C>T were found in the affected individual from family 1 (F1_P1) and in one asymptomatic relative (F1_R6) aged 45 years old (**Table 2**, **Figure 2A**). The variant *RTEL1* c.2579C>T was identified in the affected member (F9_P1) and the only relative (F9_R1) who was available for sequencing from family 9 (**Table 2**, **Figure 2A**). The damaging effect of these three variants was strongly supported by population frequency data and prediction scores (**Figure 2C**), and phenotype-genotype correlation considering both IPF and severe TL shortening (**Table 3**, **Figure 2A**). However, no other symptoms were observed, and the available evidence is currently insufficient to determine its role in the disease. For that reason, they were classified as VUS by the algorithms although we considered that they might be relevant in the disease.

Variants identified in non-telomere related genes (*SFTPA1* and *SPDL1*) and telomere-related genes (*RTEL1* and *TERT*) were classified as VUS as there was conflicting evidence supporting pathogenity (**Table 2**, **Supplemental Figure 2A**). All these variants resulted in nonsynonymous amino acid substitutions, were absent from local population controls and were present at extremely low frequency in gnomAD (MAF<0.0001) (**Supplemental Figure 2D**). The variant *SFTPA2* c.482G>A was identified in affected patient F11_P1 (Family 11). The variant clustered with previously reported pathogenic variants on the gene affecting the carbohydrate recognition domain (**Table 2**, **Supplementary Figure 2B**). However, in silico prediction scores supported a B effect (**Supplementary Figure 2D**). The splicing defect variant c.892-2A>G in *SPDL1* was only identified in F6_P1 (Family 6). It was classified as pathogenic according to SQUIRLS score (>0.9) (**Supplementary Figure 2C**). However, the variant has been previously classified as LB by ClinVar (**Table 2**, **Supplementary Figure 2D**). The variant *RTEL1* c.3470C>A was identified in the affected member F8_P1 and in the unaffected relatives F8_R1, F8_R3, and F8_6 (Family 8) (**Table 2**, **Supplementary Figure 2A**). The variant was extremely rare but was not associated with TL shortening in the affected case (**Supplementary Figure 2A**). *TERT* c.2885G>A was identified in the affected members F7_P1, F7_P2, and the unaffected relatives F7_R1, F7_R2, and F7_R3 (Family 7) (**Table 2**, **Supplementary Figure 2A**). Although the variant is very rare (MAF<0.0001), the CADD score does not support pathogenicity and the variant has been previously classified as LB by ClinVar (**Supplementary Figure 2D**). In the affected member F7_P1, cryptogenic cirrhosis (diagnosed at 30 years old) preceded IPF diagnosis, although TL measures were not available to confirm a telomere syndrome (**Supplementary Figure 2A**).

Further details of the clinical characteristics of patients are summarised in **Table 3**.

1. **Coverage of genes from Panel A in three commercial whole-exome enrichment solutions**

To evaluate the coverage of genes included in Panel A, we compared three commercial whole-exome enrichment solutions: Illumina DNA Prep with Enrichment (Illumina Inc., San Diego, CA, USA) (n=19), Illumina DNA Prep with Exome 2.5 Enrichment (Illumina Inc.) (n=20), and SureSelect XT HS2 Human All Exon V8 (Agilent Technologies, Santa Clara, CA, USA) (n=13).

For this aim, we used samples from affected patients from this study whose libraries were prepared using Illumina DNA Prep with Enrichment protocol following the manufacturer´s recommendations for each enrichment protocol. For the other exome enrichment solutions, we used data from unrelated donors from the same population from other studies.

Depth of sequencing was obtained from BAM files using the command-line tool Mosdepth (1). Coverage metrics were then calculated for each gene considering on-target-regions (exonic regions ± 10 bp) and off-target regions (exonic and intronic regions). Results of these analysis are provided in **Supplementary Figure 3**.

1. **Validation of results in FPF using automated bioinformatic prioritization tools.**

Given that all candidate variants were exclusively detected focusing on Panel A genes, we then evaluated if two of the best performing automated prioritization tools (<https://github.com/genomicsITER/benchmark-germline-variants-prioritizers>) ranked on the top of the list the same candidate variants when searching across the whole exome data. For this, the candidate P, LP, and VUS variants identified using the first-tier approach (Panel A) were used as the ground truth for the comparison. Exomiser v13.0.0 (<https://github.com/exomiser/Exomiser>) and Franklin (<https://franklin.genoox.com/clinical-db/home>) were used following a phenotype-driven approach. These tools can identify potential causative variants in genes that match the patient's phenotype as recorded using Human Phenotype Ontology (HPO) terms (**Supplementary Table 5**). This matching process involves referencing knowledge from gene-disease databases, model organism data, and the application of criteria outlined in the ACMG guidelines. The ability of both tools to assist in the accurate identification of potentially deleterious variants (variants in Panel A) was evaluated under two scenarios: 1) the candidate variant is ranked first, and 2) the candidate variant is ranked among the top five prioritized variants. Sensitivity (i.e., the percentage of patients carrying the candidate P/LP/VUS variants) and positive predictive value (i.e., the percentage of prioritized variants that resulted in a positive diagnosis) were calculated for each option.

When focusing only on the top-first ranked variants, Franklin correctly prioritized the candidate variant (P/LP/VUS) in 50% of the cases while Exomiser did it in 41.7% of the cases. If only P/LP variants were considered, Franklin correctly prioritized the candidate variant in 75% of the cases while Exomiser did it in 50%. In a comparison considering that the candidate variants (P/LP/VUS) were among the top-five ranked, Exomiser provided better results since it prioritized the candidate variants in 75% of the cases, while Franklin did it in 66.7% of the occasions. However, when only P/LP variants were considered, Franklin performed better, prioritizing the causative variants in 100% of the cases, in comparison with Exomiser, which prioritized the candidate variant in 75% of the cases. The combination of results from both tools was able to identify all candidate variants in the affected subjects (**Figure 3**).

**Supplementary Tables**

**Supplementary Table 1. Genes related to interstitial lung diseases, IPF susceptibility genes, and telomere maintenance included in Panels B and C.**

| **Gene** | **Panel** | **Category** | **Phenotype (OMIM)** | **References** |
| --- | --- | --- | --- | --- |
| *MUC5B* | B | IPF GWAS | Susceptibility to IPF | (2–7) |
| *FAM13A* | B | IPF GWAS | Susceptibility to IPF | (5,6) |
| *DSP* | B | IPF GWAS | Arrhythmogenic right ventricular dysplasia 8; Cardiomyopathy, dilated, with woolly hair and keratoderma; Dilated cardiomyopathy with woolly hair, keratoderma, and tooth agenesis; Epidermolysis bullosa, lethal acantholytic; Keratosis palmoplantaris striata II; Susceptibility to IPF | (5,6) |
| *OBFC1* | B | IPF GWAS | Cerebroretinal microangiopathy with calcifications and cysts 2; Susceptibility to IPF | (5) |
| *ATP11A* | B | IPF GWAS | Auditory neuropathy, autosomal dominant 2; Leukodystrophy, hypomyelinating, 24; Deafness, autosomal dominant 84; Susceptibility to IPF | (5,6) |
| *DPP9* | B | IPF GWAS | Hatipoglu immunodeficiency syndrome; Susceptibility to IPF | (5) |
| *TOLLIP* | B | IPF GWAS | Susceptibility to IPF | (4) |
| *MDGA2* | B | IPF GWAS | Susceptibility to IPF | (4) |
| *SPPL2C* | B | IPF GWAS | Susceptibility to IPF | (4) |
| *TGFB1* | B | IPF GWAS | Cystic fibrosis lung disease, modifier of; Camurati-Engelmann disease; Inflammatory bowel disease, immunodeficiency, and encephalopathy; Susceptibility to IPF | (8) |
| *IL1RN* | B | IPF GWAS | Gastric cancer risk after *H. pylori* infection; Microvascular complications of diabetes 4; Chronic recurrent multifocal osteomyelitis 2, with periostitis and pustulosis; Interleukin 1 receptor antagonist deficiency; Susceptibility to IPF | (9,10) |
| *CXCL8* | B | IPF GWAS | Susceptibility to IPF | (11) |
| *TLR3* | B | IPF GWAS | HIV1 infection, resistance to; Immunodeficiency 83, susceptibility to viral infections; Susceptibility to IPF | (12) |
| *CDKN1A* | B | IPF GWAS | Susceptibility to IPF | (13) |
| *TP53* | B | IPF GWAS | Adrenocortical carcinoma, pediatric; Basal cell carcinoma 7; Choroid plexus papilloma; Colorectal cancer; Glioma susceptibility 1; Osteosarcoma; Bone marrow failure syndrome 5; Breast cancer, somatic; Hepatocellular carcinoma, somatic; Li-Fraumeni syndrome; Nasopharyngeal carcinoma, somatic; Pancreatic cancer, somatic; Susceptibility to IPF | (13) |
| *ELMOD2* | B | IPF GWAS | Susceptibility to IPF | (14) |
| *MAD1L1* | B | IPF GWAS | Lymphoma, B-cell, somatic; Mosaic variegated aneuploidy syndrome 7 with inflammation and tumor predisposition; Prostate cancer, somatic | (6) |
| *DEPTOR* | B | IPF GWAS | Susceptibility to IPF | (6) |
| *HECTD2* | B | IPF GWAS | Susceptibility to IPF | (6) |
| *IVD* | B | IPF GWAS | Isovaleric acidemia | (6) |
| *AKAP13* | B | IPF GWAS | Susceptibility to IPF | (6) |
| *KANSL1* | B | IPF GWAS | Koolen-De Vries syndrome; Susceptibility to IPF | (6) |
| *GPR157* | B | IPF GWAS | Susceptibility to IPF | (15) |
| *DNAJB4* | B | IPF GWAS | Congenital myopathy 21 with early respiratory failure/ Susceptibility to IPF | (15) |
| *GIPC2* | B | IPF GWAS | Susceptibility to IPF | (15) |
| *RAPGEF2* | B | IPF GWAS | Epilepsy, familial adult myoclonic, 7: Susceptibility to IPF | (15) |
| *PSKH1* | B | IPF GWAS | Susceptibility to IPF | (15) |
| *FUT6* | B | IPF GWAS | Fucosyltransferase 6 deficiency; Susceptibility to IPF | (15) |
| *MOB2* | B | IPF GWAS | Susceptibility to IPF | (15) |
| *ACTRT3* | B | IPF GWAS | Susceptibility to IPF | (15) |
| *ARHGDIG* | B | IPF GWAS | Susceptibility to IPF | (15) |
| *CRHR1* | B | IPF GWAS | Susceptibility to IPF | (15) |
| *GMEB2* | B | IPF GWAS | Susceptibility to IPF | (15) |
| *ACD* | B | Interstitial lung disease | Inherited bone marrow failure (telomeres)/ Dyskeratosis congenita, autosomal dominant 6; Dyskeratosis congenita, autosomal recessive 7 | (16,17) |
| *NHP2* | B | Interstitial lung disease | Susceptibility to IPF and Høyeraal–Hreidarsson syndrome; Dyskeratosis congenita, autosomal recessive 2 | (18) |
| *NOP10* | B | Interstitial lung disease | Cataracts, hearing impairment, nephrotic syndrome, and enterocolitis 2; Dyskeratosis congenita, autosomal recessive 1; Susceptibility to IPF and/or bone marrow failure syndrome, telomere-related, 9 | (19) |
| *AP3B1* | B | Interstitial lung disease | Hermansky-Pudlak syndrome 2 | (20) |
| *CSF2RA* | B | Interstitial lung disease | Surfactant metabolism dysfunction, pulmonary, 4 | (20) |
| *CSF2RB* | B | Interstitial lung disease | Surfactant metabolism dysfunction, pulmonary, 5 | (20) |
| *CTC1* | B | Interstitial lung disease | Cerebroretinal microangiopathy with calcifications and cysts | (20) |
| *FAM111B* | B | Interstitial lung disease | Poikiloderma, hereditary fibrosing, with tendon contractures, myopathy, and Susceptibility to IPF | (20) |
| *MARS1* | B | Interstitial lung disease | Trichothiodystrophy 9, nonphotosensitive; Charcot-Marie-Tooth disease, axonal, type 2U; Interstitial lung and liver disease; Spastic paraplegia 70, autosomal recessive | (20) |
| *NKX2-1* | B | Interstitial lung disease | Thyroid cancer, nonmedullary, 1; Chorea, hereditary benign; Choreoathetosis, hypothyroidism, and neonatal respiratory distress | (20) |
| *POT1* | B | Interstitial lung disease | Cerebroretinal microangiopathy with calcifications and cysts 3; Susceptibility to IPF and/or bone marrow failure syndrome, telomere-related, 8; Glioma susceptibility 9; Melanoma, cutaneous malignant, susceptibility to, 10 | (20) |
| *RNF168* | B | Interstitial lung disease | RIDDLE syndrome | (20) |
| *SAMD9L* | B | Interstitial lung disease | Ataxia-pancytopenia syndrome; Monosomy 7 myelodysplasia and leukemia syndrome 1; Spinocerebellar ataxia 49 | (20) |
| *SFTPB* | B | Interstitial lung disease | Surfactant metabolism dysfunction, pulmonary, 1 | (21) |
| *SFTPD* | B | Interstitial lung disease | . | (20) |
| *WRAP53* | B | Interstitial lung disease | Dyskeratosis congenita, autosomal recessive | (20) |
| *HLA-DPB1* | B | Interstitial lung disease | Chronic beryllium disease (CBD)/Beryllium disease, chronic, susceptibility to | (5) |
| *ECM1* | B | Interstitial lung disease | Lipoid proteinosis/Urbach-Wiethe disease | (22) |
| *FBN1* | B | Interstitial lung disease | Acromicric dysplasia; Ectopia lentis, familial; Geleophysic dysplasia 2; Marfan lipodystrophy syndrome; Marfan syndrome; MASS syndrome; Stiff skin syndrome; Weill-Marchesani syndrome 2, dominant | (23) |
| *FLCN* | B | Interstitial lung disease | Birt-Hogg-Dube syndrome; Colorectal cancer, somatic; Pneumothorax, primary spontaneous; Renal carcinoma, chromophobe, somatic | (24) |
| *GATA2* | B | Interstitial lung disease | Alveolar proteinosis/Leukemia, acute myeloid, susceptibility to; Myelodysplastic syndrome, susceptibility to; Emberger syndrome; Immunodeficiency 21 | (25) |
| *GBA* | B | Interstitial lung disease | Lewy body dementia, susceptibility to; Parkinson disease, late-onset, susceptibility to; Gaucher disease, perinatal lethal; Gaucher disease, type I; Gaucher disease, type II; Gaucher disease, type III; Gaucher disease, type IIIC | (26) |
| *GLA* | B | Interstitial lung disease | Fabry disease; Fabry disease, cardiac variant | (27) |
| *HPS1* | B | Interstitial lung disease | Hermansky–Pudlak syndrome 1 | (28) |
| *HPS2* | B | Interstitial lung disease | Hermansky–Pudlak syndrome 2 | (28) |
| *HPS3* | B | Interstitial lung disease | Hermansky–Pudlak syndrome 3 | (28) |
| *HPS4* | B | Interstitial lung disease | Hermansky–Pudlak syndrome 4 | (28) |
| *NF1* | B | Interstitial lung disease | Leukemia, juvenile myelomonocytic; Neurofibromatosis-Noonan syndrome; Neurofibromatosis, familial spinal; Neurofibromatosis, type 1; Watson syndrome | (29) |
| *SLC34A2* | B | Interstitial lung disease | Pulmonary alveolar microlithiasis | (30) |
| *SLC7A7* | B | Interstitial lung disease | Lysinuric protein intolerance | (31) |
| *SMPD1* | B | Interstitial lung disease | Niemann-Pick disease, type A; Niemann-Pick disease, type B | (32) |
| *TSC1* | B | Interstitial lung disease | Focal cortical dysplasia, type II, somatic; Lymphangioleiomyomatosis; Tuberous sclerosis-1 | (33) |
| *FARSA* | B | Interstitial lung disease | Rajab interstitial lung disease with brain calcifications 2 | (34) |
| *FARSB* | B | Interstitial lung disease | Rajab interstitial lung disease with brain calcifications 1 | (35) |
| *ITGA3* | B | Interstitial lung disease | Interstitial lung disease, nephrotic syndrome, and epidermolysis bullosa, congenital | (36) |
| *TSC2* | B | Interstitial lung disease | Focal cortical dysplasia, type II, somatic; Lymphangioleiomyomatosis, somatic; Tuberous sclerosis-2 | (33) |
| *ACTL6A* | C | Telomere maintenance Ontology term | . |  |
| *ACTR8* | C | Telomere maintenance Ontology term | . |  |
| *ACYP1* | C | Telomere length GWAS | . | (37) |
| *ACYP2* | C | Telomere length GWAS | . | (38) |
| *APEX1* | C | Telomere maintenance Ontology term | . |  |
| *ATM* | C | Telomere length GWAS | Breast cancer, susceptibility to; Ataxia-telangiectasia; Lymphoma, B-cell non-Hodgkin, somatic; Lymphoma, mantle cell, somatic; T-cell prolymphocytic leukemia, somatic | (39) |
| *ATP8B4* | C | Telomere length GWAS | . |  |
| *ATR* | C | Telomere maintenance Ontology term | Cutaneous telangiectasia and cancer syndrome, familial; Seckel syndrome 1 |  |
| *ATRX* | C | Telomere maintenance Ontology term | Alpha-thalassemia myelodysplasia syndrome, somatic; Alpha-thalassemia/impaired intellectual development syndrome; Intellectual disability-hypotonic facies syndrome, X-linked |  |
| *AURKB* | C | Telomere maintenance Ontology term | . |  |
| *BANP* | C | Telomere length GWAS | . | (37) |
| *BCL2L15* | C | Telomere length GWAS | . | (37) |
| *BLM* | C | Telomere maintenance Ontology term | Bloom syndrome |  |
| *BRCA2* | C | Telomere maintenance Ontology term | Breast cancer, male, susceptibility to; Breast-ovarian cancer, familial, 2; Glioblastoma 3; Medulloblastoma; Pancreatic cancer 2; Prostate cancer; Fanconi anemia, complementation group D1; Wilms tumor |  |
| *CARMIL1* | C | Telomere length GWAS | . | (39) |
| *CCNE1* | C | Telomere maintenance Ontology term | . |  |
| *CCNE2* | C | Telomere maintenance Ontology term | . |  |
| *CCT2* | C | Telomere maintenance Ontology term | . |  |
| *CCT3* | C | Telomere maintenance Ontology term | . |  |
| *CCT4* | C | Telomere maintenance Ontology term | . |  |
| *CCT5* | C | Telomere maintenance Ontology term | Neuropathy, hereditary sensory, with spastic paraplegia |  |
| *CCT6A* | C | Telomere maintenance Ontology term | . |  |
| *CCT7* | C | Telomere maintenance Ontology term | . |  |
| *CCT8* | C | Telomere maintenance Ontology term | . |  |
| *CLEC18C* | C | Telomere length GWAS | . | (37) |
| *CSNK2A2* | C | Telomere length GWAS | . | (40) |
| *CTNNB1* | C | Telomere maintenance Ontology term | Colorectal cancer, somatic; Exudative vitreoretinopathy 7; Hepatocellular carcinoma, somatic; Medulloblastoma, somatic; Neurodevelopmental disorder with spastic diplegia and visual defects; Ovarian cancer, somatic; Pilomatricoma, somatic |  |
| *CXCR4* | C | Telomere length GWAS | Myelokathexis, isolated; WHIM syndrome 1 | (38) |
| *CXXC5* | C | Telomere length GWAS | . | (37) |
| *DCAF4* | C | Telomere length GWAS | . | (39) |
| *DCLRE1A* | C | Telomere maintenance Ontology term | . |  |
| *DCLRE1B* | C | Telomere maintenance Ontology term | Dyskeratosis congenita, autosomal recessive 8 |  |
| *DCLRE1C* | C | Telomere maintenance Ontology term | Omenn syndrome; Severe combined immunodeficiency, Athabascan type |  |
| *DCP2* | C | Telomere maintenance Ontology term | . |  |
| *DHX36* | C | Telomere maintenance Ontology term | . |  |
| *DNA2* | C | Telomere maintenance Ontology term | Seckel syndrome 8; Progressive external ophthalmoplegia with mitochondrial DNA deletions, autosomal dominant 6 |  |
| *EID3* | C | Telomere maintenance Ontology term | . |  |
| *ERCC1* | C | Telomere maintenance Ontology term | Cerebrooculofacioskeletal syndrome 4 |  |
| *ERCC4* | C | Telomere maintenance Ontology term | Fanconi anemia, complementation group Q; Xeroderma pigmentosum, group F; Xeroderma pigmentosum, type F/Cockayne syndrome; XFE progeroid syndrome |  |
| *EXO1* | C | Telomere maintenance Ontology term | . |  |
| *EXOSC10* | C | Telomere maintenance Ontology term | . |  |
| *FBXO4* | C | Telomere maintenance Ontology term | . |  |
| *FEN1* | C | Telomere maintenance Ontology term | . |  |
| *GAB3* | C | Telomere length GWAS | . | (37) |
| *GAR1* | C | Telomere maintenance Ontology term | . |  |
| *GNL3* | C | Telomere maintenance Ontology term | . |  |
| *GNL3L* | C | Telomere maintenance Ontology term | . |  |
| *HDAC8* | C | Telomere maintenance Ontology term | Cornelia de Lange syndrome 5 |  |
| *HMBOX1* | C | Telomere maintenance Ontology term | . |  |
| *HNRNPA1* | C | Telomere maintenance Ontology term | Inclusion body myopathy with early-onset Paget disease without frontotemporal dementia 3; Myopathy, distal, 3; Amyotrophic lateral sclerosis 20 |  |
| *HNRNPA2B1* | C | Telomere maintenance Ontology term | Inclusion body myopathy with early-onset Paget disease with or without frontotemporal dementia 2; Oculopharyngeal muscular dystrophy 2 |  |
| *HNRNPC* | C | Telomere maintenance Ontology term | . |  |
| *HNRNPD* | C | Telomere maintenance Ontology term | . |  |
| *HNRNPU* | C | Telomere maintenance Ontology term | Developmental and epileptic encephalopathy 54 |  |
| *HSP90AA1* | C | Telomere maintenance Ontology term | . |  |
| *HSP90AB1* | C | Telomere maintenance Ontology term | . |  |
| *HSPA1A* | C | Telomere length GWAS | . | (37) |
| *HUS1* | C | Telomere maintenance Ontology term | . |  |
| *HUS1B* | C | Telomere maintenance Ontology term | . |  |
| *INO80* | C | Telomere maintenance Ontology term | . |  |
| *INO80D* | C | Telomere maintenance Ontology term | . |  |
| *INO80E* | C | Telomere maintenance Ontology term | . |  |
| *KBTBD7* | C | Telomere length GWAS | . | (37) |
| *MAD2L2* | C | Telomere maintenance Ontology term | Fanconi anemia, complementation group V |  |
| *MAP2K7* | C | Telomere maintenance Ontology term | . |  |
| *MAP3K4* | C | Telomere maintenance Ontology term | . |  |
| *MAPK1* | C | Telomere maintenance Ontology term | Noonan syndrome 13 |  |
| *MAPK15* | C | Telomere maintenance Ontology term | . |  |
| *MAPK3* | C | Telomere maintenance Ontology term | . |  |
| *MAPKAPK5* | C | Telomere maintenance Ontology term | Neurocardiofaciodigital syndrome |  |
| *MCRS1* | C | Telomere maintenance Ontology term | . |  |
| *MOB1B* | C | Telomere length GWAS | . | (39) |
| *MPHOSPH6* | C | Telomere length GWAS | . | (39) |
| *MYC* | C | Telomere maintenance Ontology term | Burkitt lymphoma, somatic |  |
| *NABP2* | C | Telomere maintenance Ontology term | . |  |
| *NAT10* | C | Telomere maintenance Ontology term | . |  |
| *NBN* | C | Telomere maintenance Ontology term | Aplastic anemia; Leukemia, acute lymphoblastic; Nijmegen breakage syndrome |  |
| *NEK2* | C | Telomere maintenance Ontology term | Retinitis pigmentosa 67 |  |
| *NEK7* | C | Telomere maintenance Ontology term | . |  |
| *NKX2-3* | C | Telomere length GWAS | . | (37) |
| *NOC3L* | C | Telomere length GWAS | . | (37) |
| *NSMCE1* | C | Telomere maintenance Ontology term | . |  |
| *NSMCE2* | C | Telomere maintenance Ontology term | Seckel syndrome 10 |  |
| *NSMCE4A* | C | Telomere maintenance Ontology term | . |  |
| *P3H2* | C | Telomere length GWAS | Myopia, high, with cataract and vitreoretinal degeneration | (37,39) |
| *PAPSS1* | C | Telomere length GWAS | . | (39) |
| *PARP1* | C | Telomere length GWAS | . | (39) |
| *PARP3* | C | Telomere maintenance Ontology term | . |  |
| *PCNA* | C | Telomere maintenance Ontology term | Ataxia-telangiectasia-like disorder 2 |  |
| *PIF1* | C | Telomere maintenance Ontology term | . |  |
| *PINX1* | C | Telomere maintenance Ontology term | . |  |
| *PKIB* | C | Telomere maintenance Ontology term | . |  |
| *PML* | C | Telomere maintenance Ontology term | Leukemia, acute promyelocytic, PML/RARA type |  |
| *PNKP* | C | Telomere maintenance Ontology term | Charcot-Marie-Tooth disease, type 2B2; Ataxia-oculomotor apraxia 4; Microcephaly, seizures, and developmental delay |  |
| *PPP1R10* | C | Telomere maintenance Ontology term | . |  |
| *PRKCQ* | C | Telomere maintenance Ontology term | . |  |
| *PRKDC* | C | Telomere maintenance Ontology term | Immunodeficiency 26, with or without neurologic abnormalities |  |
| *PRRC2A* | C | Telomere length GWAS | . | (39) |
| *PTGES3* | C | Telomere maintenance Ontology term | . |  |
| *PXK* | C | Telomere length GWAS | . | (41) |
| *RAD50* | C | Telomere maintenance Ontology term | Nijmegen breakage syndrome-like disorder |  |
| *RAD51* | C | Telomere maintenance Ontology term | Breast cancer, susceptibility to; Fanconi anemia, complementation group R; Mirror movements 2 |  |
| *RAD51C* | C | Telomere maintenance Ontology term | Breast-ovarian cancer, familial, susceptibility to, 3; Fanconi anemia, complementation group O |  |
| *RAD51D* | C | Telomere maintenance Ontology term | Breast-ovarian cancer, familial, susceptibility to, 4 |  |
| *RECQL4* | C | Telomere maintenance Ontology term | Baller-Gerold syndrome; RAPADILINO syndrome; Rothmund-Thomson syndrome, type 2 |  |
| *RFC1* | C | Telomere maintenance Ontology term | Cerebellar ataxia, neuropathy, and vestibular areflexia syndrome |  |
| *RFWD3* | C | Telomere length GWAS | Fanconi anemia, complementation group W | (39) |
| *RIF1* | C | Telomere maintenance Ontology term | . |  |
| *RPA1* | C | Telomere maintenance Ontology term | Susceptibility to IPF and/or bone marrow failure syndrome, telomere-related, 6 |  |
| *RPA2* | C | Telomere maintenance Ontology term | . |  |
| *RPA3* | C | Telomere maintenance Ontology term | . |  |
| *RUVBL1* | C | Telomere maintenance Ontology term | . |  |
| *RUVBL2* | C | Telomere maintenance Ontology term | . |  |
| *SAMHD1* | C | Telomere length GWAS | Chilblain lupus 2; Aicardi-Goutieres syndrome 5 | (37) |
| *SENP7* | C | Telomere length GWAS | . | (39) |
| *SETBP1* | C | Telomere length GWAS | Intellectual developmental disorder, autosomal dominant 29; Schinzel-Giedion midface retraction syndrome | (37) |
| *SIRT6* | C | Telomere maintenance Ontology term | . |  |
| *SLC2A2* | C | Telomere length GWAS | Diabetes mellitus, noninsulin-dependent; Fanconi-Bickel syndrome | (37) |
| *SLX1A* | C | Telomere maintenance Ontology term | . |  |
| *SLX4* | C | Telomere maintenance Ontology term | Fanconi anemia, complementation group P |  |
| *SMARCAL1* | C | Telomere maintenance Ontology term | Schimke immunoosseous dysplasia |  |
| *SMC5* | C | Telomere maintenance Ontology term | Atelis syndrome 2 |  |
| *SMC6* | C | Telomere maintenance Ontology term | . |  |
| *SMG1* | C | Telomere maintenance Ontology term | . |  |
| *SMG5* | C | Telomere maintenance Ontology term | . |  |
| *SMG6* | C | Telomere maintenance Ontology term | . |  |
| *SP100* | C | Telomere maintenance Ontology term | . |  |
| *SRC* | C | Telomere maintenance Ontology term | Thrombocytopenia 6; Colon cancer, advanced, somatic |  |
| *STMN3* | C | Telomere length GWAS | . | (39) |
| *TCP1* | C | Telomere maintenance Ontology term | . |  |
| *TELO2* | C | Telomere maintenance Ontology term | You-Hoover-Fong syndrome |  |
| *TEP1* | C | Telomere maintenance Ontology term | . |  |
| *TERF2IP* | C | Telomere maintenance Ontology term | . |  |
| *TFIP11* | C | Telomere maintenance Ontology term | . |  |
| *TFPT* | C | Telomere maintenance Ontology term | . |  |
| *TNKS* | C | Telomere maintenance Ontology term | . |  |
| *TNKS1BP1* | C | Telomere maintenance Ontology term | . |  |
| *TNKS2* | C | Telomere maintenance Ontology term | . |  |
| *TNP03* | C | Telomere length GWAS | . | (37) |
| *TPRKB* | C | Telomere maintenance Ontology term | Galloway-Mowat syndrome 5 |  |
| *TYMP* | C | Telomere length GWAS | Mitochondrial DNA depletion syndrome 1 (MNGIE type) | (37) |
| *TYMS* | C | Telomere length GWAS | Dyskeratosis congenita, digenic | (37) |
| *UCHL5* | C | Telomere maintenance Ontology term | . |  |
| *UPF1* | C | Telomere maintenance Ontology term | . |  |
| *USP7* | C | Telomere maintenance Ontology term | Hao-Fountain syndrome |  |
| *VSIG4* | C | Telomere length GWAS | . | (37) |
| *WRN* | C | Telomere maintenance Ontology term | Werner syndrome |  |
| *XRCC1* | C | Telomere maintenance Ontology term | Spinocerebellar ataxia, autosomal recessive 26 |  |
| *XRCC3* | C | Telomere maintenance Ontology term | Breast cancer, susceptibility to; Melanoma, cutaneous malignant, 6 |  |
| *XRCC5* | C | Telomere maintenance Ontology term | . |  |
| *XRCC6* | C | Telomere maintenance Ontology term | . |  |
| *XRN1* | C | Telomere maintenance Ontology term | . |  |
| *YLPM1* | C | Telomere maintenance Ontology term | . |  |
| *YY1* | C | Telomere maintenance Ontology term | Gabriele-de Vries syndrome |  |
| *ZBTB46* | C | Telomere length GWAS | . | (39) |
| *ZBTB48* | C | Telomere maintenance Ontology term | . |  |
| *ZMYM4* | C | Telomere length GWAS | . | (37) |
| *ZNF209* | C | Telomere length GWAS | . | (39) |
| *ZNF257* | C | Telomere length GWAS | . | (37) |
| *ZNF365* | C | Telomere maintenance Ontology term | Nephrolithiasis, uric acid, susceptibility to |  |
| *ZNF827* | C | Telomere maintenance Ontology term | . |  |
| *ZSCAN4* | C | Telomere maintenance Ontology term | . |  |
| *TEN1* | C | Telomere maintenance Ontology term | . |  |
| *TERF2* | C | Telomere maintenance Ontology term | . |  |
| *TERF1* | C | Telomere maintenance Ontology term | . |  |
| *STING1* | C | Telomere maintenance Ontology term | STING-associated vasculopathy, infantile-onset |  |
| *USB1* | C | Telomere maintenance Ontology term | Poikiloderma with neutropenia |  |

| Supplementary Table 2. Sanger sequencing details of gene regions containing the prioritized variants from Panel A and B (*SFTPD*). Primers sequences and PCR conditions used for amplification and sequencing are also indicated. | | | | | |
| --- | --- | --- | --- | --- | --- |
| Gene | **Variant** | **Primer sequences**  **(5´-3’)** | **Product length (bp)** | **Tm (ºC)** | **MgCl_2_**  **concentration (mM)** |
| *RTEL1* | NM_001283009.2:c.3470C>A | TGTGCCGGGTCTGATTGAA  AGATCTTGCTCTGGGTCTTCC | 337 | 57 | 2 |
| *RTEL1* | NM_001283009.2:c.2920C>T | CAGGACTACAAGGGTTCCGA  CATAGGGGAACAGAGAGGCG | 498 | 57 | 2 |
| *RTEL1* | NM_001283009.2:c.2579C>T | CCCTGGACCTGCTCTTACAA  AGAAACCACACACTTGAGCC | 498 | 57 | 2 |
| *SFTPA2* | NM_001098668.4:c.482G>A | ACATCTCCACACACTGCTCTT  AGTTCTCTGCCTGTCTTGCT | 575 | 57 | 2 |
| *TINF2* | NM_001099274.3:c.1108C>T | CCTTCCCACTCACCTTTCCT  CCAGTTGACTTGCCTGCCA | 400 | 59 | 1.5 |
| *RTEL1* | NM_001283009.2:c.2935C>T | TTTGCTGAGGACCCCAAGAA  CATAGGGGAACAGAGAGGCG | 441 | 59 | 1.5 |
| *NAF1* | NM_138386.3:c.1104T>G | GGAAATCCTGAAGTCTCCTGAG  TCTGACTTGCTGGGTTCTCT | 396 | 59 | 2 |
| *SPDL1* | NM_017785.5:c.892-2A>G | CACAGCCATGCAAGTCAGTT  CACCATTCTTCTGCTCCAACA | 357 | 60 | 1.5 |
| *TERT* | NM_198253.3:c.2885G>A | CACACACACACATACTTGCG  GGAGTTTGGTCATGCAGAGTC | 398 | 60 | 1.5 |
| *SFTPD* | NM_003019.5:c.178C>T | CTCAGCACAGCCACTTGTTT  GGGGCTTCTTGTCACCTCTA | 465 | 60 | 1.5 |

| **Supplementary Table 3. Fraction of bases (%) covered at 10X depth in FPF patients from this study.** | | | | | | | | | | | | | |
| --- | --- | --- | --- | --- | --- | --- | --- | --- | --- | --- | --- | --- | --- |
| **Patient** | ***ABCA3*** | ***DKC1*** | ***KIF15*** | ***NAF1*** | ***PARN*** | ***RTEL1*** | ***SFTPA2*** | ***SFTPC*** | ***SPDL1*** | ***TERC*** | ***TERT*** | ***TINF2*** | ***ZCCHC8*** |
| F5_P1 | 84 | 72 | 90 | 68 | 69 | 83 | 56 | 47 | 96 | 77 | 58 | 82 | 54 |
| F3_P1 | 85 | 70 | 89 | 70 | 69 | 82 | 56 | 48 | 93 | 77 | 59 | 81 | 54 |
| F6_P1 | 89 | 71 | 90 | 70 | 71 | 83 | 61 | 48 | 96 | 78 | 60 | 80 | 55 |
| F4_P1 | 88 | 71 | 89 | 68 | 71 | 80 | 60 | 47 | 93 | 78 | 59 | 83 | 53 |
| F2_P2 | 87 | 74 | 89 | 73 | 70 | 85 | 56 | 47 | 93 | 77 | 67 | 83 | 54 |
| F5_P2 | 85 | 70 | 88 | 69 | 69 | 81 | 55 | 47 | 93 | 74 | 57 | 79 | 51 |
| F1_P1 | 86 | 69 | 90 | 68 | 69 | 83 | 58 | 46 | 94 | 77 | 58 | 82 | 53 |
| F12_P1 | 88 | 71 | 92 | 70 | 69 | 86 | 66 | 51 | 99 | 80 | 63 | 83 | 58 |
| F12_P2 | 91 | 75 | 92 | 79 | 73 | 89 | 67 | 54 | 97 | 78 | 72 | 84 | 57 |
| F13_P1 | 87 | 70 | 91 | 76 | 70 | 85 | 63 | 48 | 97 | 78 | 63 | 83 | 56 |
| F7_P1 | 90 | 71 | 91 | 78 | 71 | 88 | 63 | 55 | 99 | 79 | 66 | 83 | 57 |
| F7_P2 | 92 | 75 | 92 | 81 | 71 | 91 | 66 | 49 | 100 | 78 | 73 | 85 | 56 |
| F8_P1 | 91 | 74 | 91 | 79 | 72 | 89 | 63 | 49 | 100 | 78 | 67 | 84 | 57 |
| F10_P1 | 87 | 72 | 91 | 71 | 71 | 86 | 60 | 48 | 96 | 78 | 63 | 82 | 55 |
| F9_P1 | 89 | 70 | 91 | 74 | 71 | 82 | 60 | 54 | 99 | 79 | 62 | 83 | 57 |
| F11_P1 | 88 | 72 | 91 | 72 | 71 | 83 | 62 | 48 | 99 | 78 | 63 | 83 | 56 |
| Mean | 87.9 | 71.7 | 90.4 | 72.9 | 70.4 | 84.8 | 60.8 | 49.1 | 96.5 | 77.8 | 63.1 | 82.5 | 55.2 |
| SD | 2.3 | 1.8 | 1.1 | 4.8 | 1.2 | 3.2 | 3.7 | 2.6 | 2.6 | 1.2 | 4.9 | 1.5 | 1.8 |
| SD, standard deviation. | | | | | | | | | | | | | |

| Supplementary Table 4. List of VUS identified in the study using Panels B and C. In the second tier, rare deleterious variants were identified in index cases using two virtual gene panels (Panels B and C). | | | | | | | | |
| --- | --- | --- | --- | --- | --- | --- | --- | --- |
| *Case ID* | ***TL***  ***(Percentile)*** | ***Gene*** | ***HGVS*** | ***Amino acid change*** | ***Functional effect*** | ***Allele frequency (gnomAD)*** | ***Zygosity*** | ***ACMG class*** |
| F8_P1 | 25-50 | *MUC5B* | NM_002458.3:c.2230G>A | p.Gly744Ser | Missense | 0.001154 | Het | VUS |
| F9_P1 | <10 | *HPS3* | NM_032383.5:c.127G>A | p.Ala43Thr | Missense | 0.000004471 | Het | VUS |
| F9_P1 | <10 | *INO80* | NM_017553.3:c.1195A>G | p.Met399Val | Missense | Absent | Het | VUS |
| F9_P1 | <10 | *TELO2* | NM_016111.4:c.983G>A | p.Arg328Gln | Missense | 0.00004517 | Het | VUS |
| F10_P1 | 25-50 | *WRAP53* | NM_001143992.2:c.187G>A | p.Val63Met | Missense | 0.0003189 | Het | VUS |
| F10_P1 | 25-50 | *SFTPD* | NM_003019.5:c.25C>A | p.Leu9Met | Missense | 0.003 | Het | VUS |
| F10_P1 | 25-50 | *GLA* | NM_000169.3:c.352C>T | p.Arg118Cys | Missense | 0.0002339 | Het | VUS |
| F11_P1 | 10-25 | *DPP9* | NM_139159.5:c.2309T>G | p.Ile770Ser | Missense | Absent | Het | VUS |
| F11_P1 | 10-25 | *FAM111B* | NM_198947.4:c.1619A>G | p.Asn540Ser | Missense | 0.0000638 | Het | VUS |
| F11_P1 | 10-25 | *ACTRT3* | NM_032487.5:c.199A>G | p.Ser67Gly | Missense | Absent | Het | VUS |
| F11_P1 | 10-25 | *TSC1* | NM_000368.5:c.2282A>G | p.Tyr761Cys | Missense | Absent | Het | VUS |
| F11_P1 | 10-25 | *LIG4* | NM_206937.2:c.1604C>G | p.Pro535Arg | Missense | Absent | Het | VUS |
| F11_P1 | 10-25 | *AKAP13* | NM_007200.5:c.7387C>T | p.Arg2463Trp | Missense | 0.00002122 | Het | VUS |
| F11_P1 | 10-25 | *MAD1L1* | NM_001013836.2:c.1697C>T | p.Ala566Val | Missense | 0.00002915 | Het | VUS |
| F1_P1 | 25-50 | *RAPGEF2* | NM_001351724.5:c.4779dup | p.Gly1594TrpfsTer6 | Frameshift | Absent | Het | VUS |
| F2_P2 | 25-50 | *MUC5B* | NM_002458.3:c.7762G>A | p.Gly2588Arg | Missense | 0.001540 | Het | VUS |
| F4_P4 | 25-50 | *AKAP13* | NM_007200.5:c.8206C>T | p.Arg2736Trp | Missense | 0.0003676 | Het | VUS |
| F6_P1 | 50-75 | *SFTPD* | NM_003019.5:c.178C>T | p.Arg2736Trp | Missense | 0.00003892 | Het | VUS |
| VUS, variants of uncertain significance; TL, telomere length; HGVS, Human Genome Variation Society; Het, heterozygous; ACMG, American College of Medical Genetics. | | | | | | | | |

| Supplementary Table 5. Human Phenotype Ontology (HPO) terms used for recording phenotypes associated with IPF in Exomiser and Franklin. | |
| --- | --- |
| Term identifier | **Term name** |
| HP:0012735 | Cough |
| HP:0031950 | Usual interstitial pneumonia |
| HP:0045051 | Decreased DLCO |
| HP:0032977 | Elevated bronchoalveolar lavage fluid neutrophil proportion |
| HP:0002206 | Pulmonary fibrosis |
| HP:0002875 | Exertional dyspnea |

**Supplementary Figures**

**Supplementary Figure 1. Pedigrees from 13 families with FPF included in the study**. Information from at least two generations was recorded and unique IDs were assigned to individuals participating in the study. Information about age and telomere length measurements (percentiles) is also provided. Y: years; p: percentile.


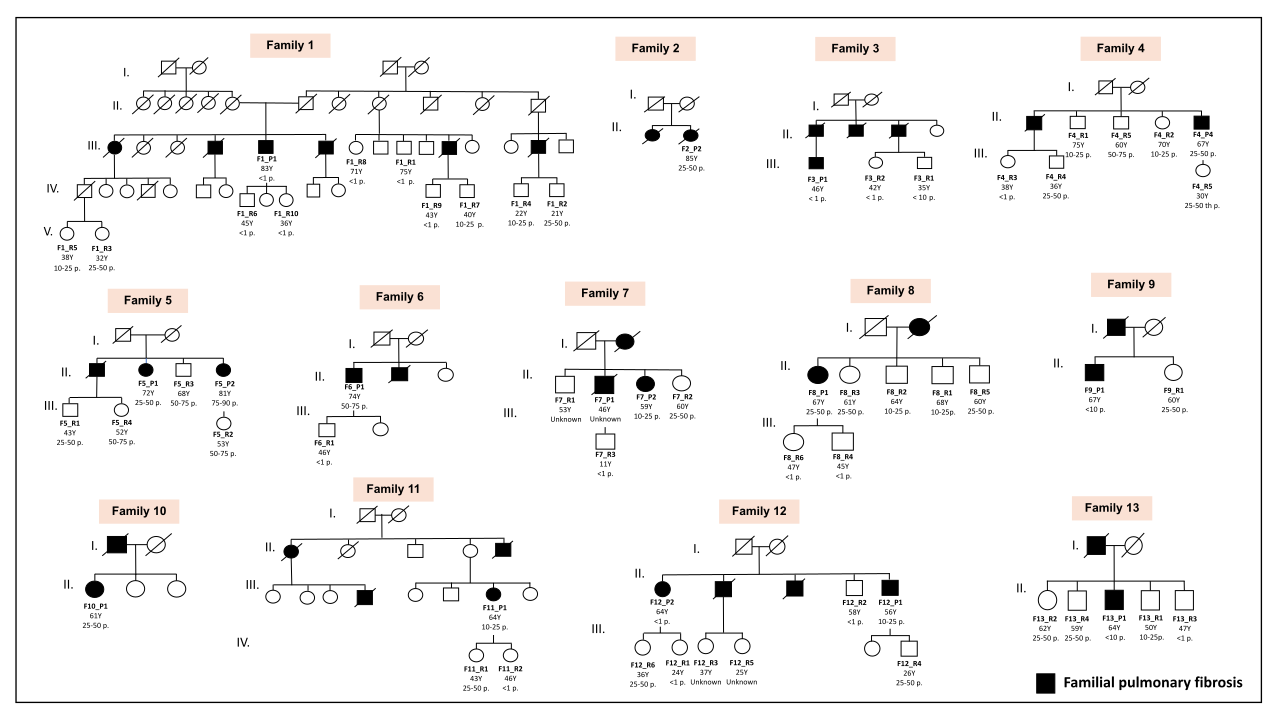


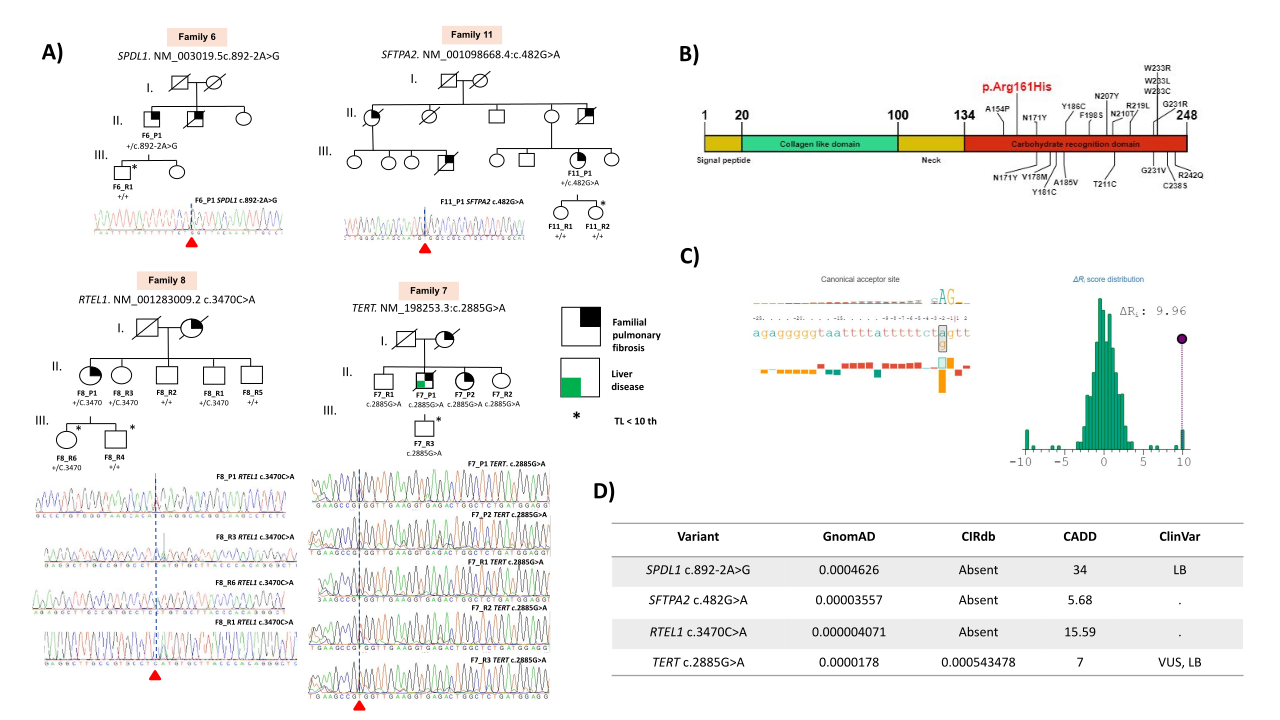
**Supplementary Figure 2.** **Pedigrees of the families carrying variants of uncertain significance in genes from Panel A**. A) *SPDL1* genotypes are shown below the sequenced individuals from family 6. Sequence electropherograms (bottom) supporting the variant c.892-2A>G are shown. *SFTPA2* genotypes are shown below the sequenced individuals from family 11. Sequence electropherograms (bottom) supporting the variant c.482G>A are shown. *RTEL1* genotypes are shown below the sequenced individuals from family 8. Sequence electropherograms (bottom) supporting the variant c.3470C>A are shown. *TERT* genotypes are shown below the sequenced individuals from family 7. Sequence electropherograms (bottom) supporting the variant c.2885G>A are shown. B) Scheme of the *SFTPA2* gene showing a novel variant (in red) which clusters with previously reported variants in patients with pulmonary fibrosis (black). C) Representation generated by SQUIRLs of predictor acceptor site for variant c.892-2A>G in *SPDL1*. D) Population frequency data, the *in silico* predictor CADD score, and ClinVar classification of candidate variants. A + symbol on the genotypes stands for the reference allele.

**Supplementary Figure 3. Proportion of bases covered on average per gene (Panel A).** A) Proportion of bases covered at 10X per gene. B) Proportion of bases covered at least 1X per gene.

**
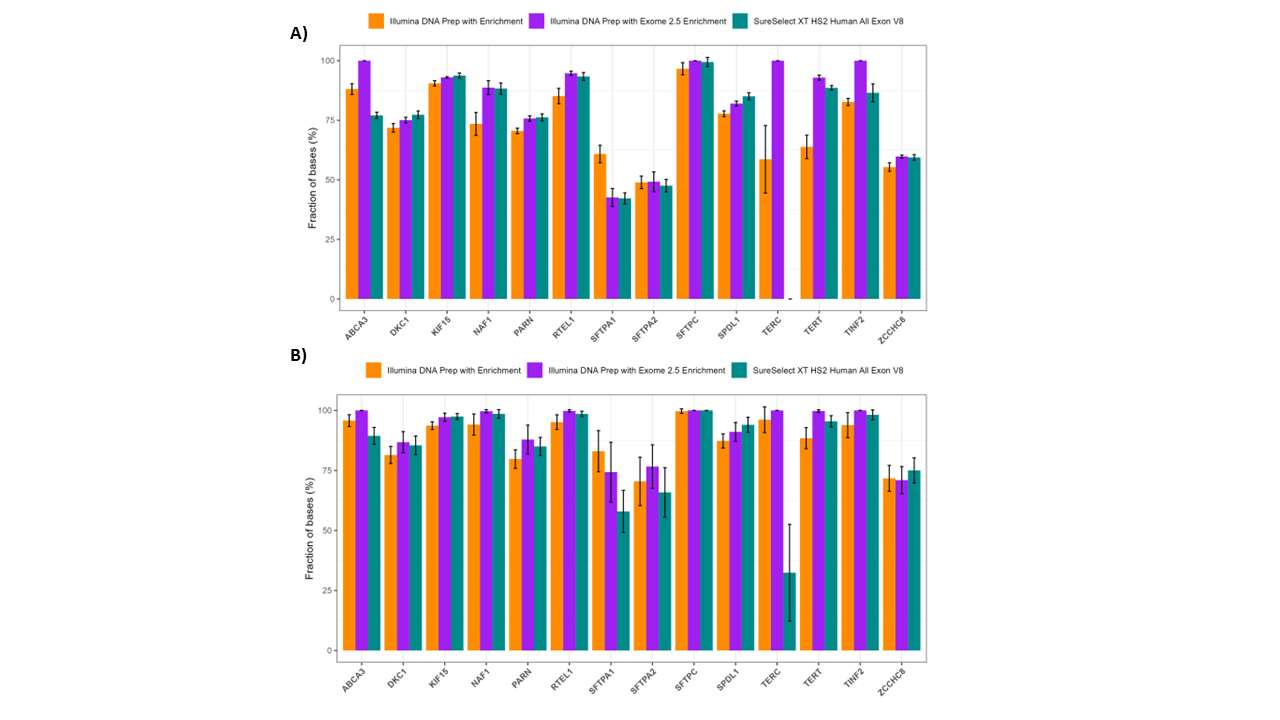
**

**Supplementary References**

1. Pedersen BS, Quinlan AR. Mosdepth: quick coverage calculation for genomes and exomes. Bioinforma Oxf Engl. 2018 Mar 1;34(5):867–8.

2. Seibold MA, Wise AL, Speer MC, Steele MP, Brown KK, Loyd JE, et al. A common MUC5B promoter polymorphism and pulmonary fibrosis. N Engl J Med. 2011 Apr 21;364(16):1503–12.

3. Peljto AL, Zhang Y, Fingerlin TE, Ma SF, Garcia JGN, Richards TJ, et al. Association between the MUC5B promoter polymorphism and survival in patients with idiopathic pulmonary fibrosis. JAMA. 2013 Jun 5;309(21):2232–9.

4. Noth I, Zhang Y, Ma SF, Flores C, Barber M, Huang Y, et al. Genetic variants associated with idiopathic pulmonary fibrosis susceptibility and mortality: a genome-wide association study. Lancet Respir Med. 2013 Jun;1(4):309–17.

5. Fingerlin TE, Murphy E, Zhang W, Peljto AL, Brown KK, Steele MP, et al. Genome-wide association study identifies multiple susceptibility loci for pulmonary fibrosis. Nat Genet. 2013 Jun;45(6):613–20.

6. Allen RJ, Guillen-Guio B, Oldham JM, Ma SF, Dressen A, Paynton ML, et al. Genome-Wide Association Study of Susceptibility to Idiopathic Pulmonary Fibrosis. Am J Respir Crit Care Med. 2020 Mar;201(5):564–74.

7. Peljto AL, Selman M, Kim DS, Murphy E, Tucker L, Pardo A, et al. The MUC5B promoter polymorphism is associated with idiopathic pulmonary fibrosis in a Mexican cohort but is rare among Asian ancestries. Chest. 2015 Feb;147(2):460–4.

8. Son JY, Kim SY, Cho SH, Shim HS, Jung JY, Kim EY, et al. TGF-β1 T869C polymorphism may affect susceptibility to idiopathic pulmonary fibrosis and disease severity. Lung. 2013 Apr;191(2):199–205.

9. Barlo NP, van Moorsel CHM, Korthagen NM, Heron M, Rijkers GT, Ruven HJT, et al. Genetic variability in the IL1RN gene and the balance between interleukin (IL)-1 receptor agonist and IL-1β in idiopathic pulmonary fibrosis. Clin Exp Immunol. 2011 Dec;166(3):346–51.

10. Korthagen NM, van Moorsel CHM, Kazemier KM, Ruven HJT, Grutters JC. IL1RN genetic variations and risk of IPF: a meta-analysis and mRNA expression study. Immunogenetics. 2012 May 1;64(5):371–7.

11. Ahn MH, Park BL, Lee SH, Park SW, Park JS, Kim DJ, et al. A promoter SNP rs4073T>A in the common allele of the interleukin 8 gene is associated with the development of idiopathic pulmonary fibrosis via the IL-8 protein enhancing mode. Respir Res. 2011 Dec 1;12(1):73.

12. O’Dwyer DN, Armstrong ME, Trujillo G, Cooke G, Keane MP, Fallon PG, et al. The Toll-like receptor 3 L412F polymorphism and disease progression in idiopathic pulmonary fibrosis. Am J Respir Crit Care Med. 2013 Dec 15;188(12):1442–50.

13. Korthagen NM, Moorsel CHM van, Barlo NP, Kazemier KM, Ruven HJT, Grutters JC. Association between Variations in Cell Cycle Genes and Idiopathic Pulmonary Fibrosis. PLOS ONE. 2012 Jan 23;7(1):e30442.

14. Hodgson U, Pulkkinen V, Dixon M, Peyrard-Janvid M, Rehn M, Lahermo P, et al. ELMOD2 is a candidate gene for familial idiopathic pulmonary fibrosis. Am J Hum Genet. 2006 Jul;79(1):149–54.

15. Partanen JJ, Häppölä P, Zhou W, Lehisto AA, Ainola M, Sutinen E, et al. Leveraging global multi-ancestry meta-analysis in the study of idiopathic pulmonary fibrosis genetics. Cell Genomics. 2022;2(10):100181.

16. Hoffman TW, van der Vis JJ, van der Smagt JJ, Massink MPG, Grutters JC, van Moorsel CHM. Pulmonary fibrosis linked to variants in the ACD gene, encoding the telomere protein TPP1. Eur Respir J. 2019 Dec;54(6):1900809.

17. Guo Y, Kartawinata M, Li J, Pickett HA, Teo J, Kilo T, et al. Inherited bone marrow failure associated with germline mutation of ACD, the gene encoding telomere protein TPP1. Blood. 2014 Oct 30;124(18):2767–74.

18. Benyelles M, O’Donohue MF, Kermasson L, Lainey E, Borie R, Lagresle-Peyrou C, et al. NHP2 deficiency impairs rRNA biogenesis and causes pulmonary fibrosis and Høyeraal–Hreidarsson syndrome. Hum Mol Genet. 2020 Apr 15;29(6):907–22.

19. Kannengiesser C, Manali ED, Revy P, Callebaut I, Ba I, Borgel A, et al. First heterozygous NOP10 mutation in familial pulmonary fibrosis. Eur Respir J. 2020 Jun;55(6):1902465.

20. Mathai SK, Schwartz DA, Borie R. 15 - Genetic Determinants of Interstitial Lung Diseases∗. In: Pyeritz RE, Korf BR, Grody WW, editors. Emery and Rimoin’s Principles and Practice of Medical Genetics and Genomics (Seventh Edition) [Internet]. Academic Press; 2020. p. 405–37. Available from: https://www.sciencedirect.com/science/article/pii/B978012812532800015X

21. Desroziers T, Prévot G, Coulomb A, Nau V, Dastot-Le Moal F, Duquesnoy P, et al. Hypomorphic pathogenic variant in SFTPB leads to adult pulmonary fibrosis. Eur J Hum Genet EJHG. 2023 Sep;31(9):1083–7.

22. Hamada T, McLean WHI, Ramsay M, Ashton GHS, Nanda A, Jenkins T, et al. Lipoid proteinosis maps to 1q21 and is caused by mutations in the extracellular matrix protein 1 gene (ECM1). Hum Mol Genet. 2002 Apr 1;11(7):833–40.

23. Judge DP, Dietz HC. Marfan’s syndrome. The Lancet. 2005 Dec 3;366(9501):1965–76.

24. Furuya M, Tanaka R, Koga S, Yatabe Y, Gotoda H, Takagi S, et al. Pulmonary Cysts of Birt-Hogg-Dubé Syndrome: A Clinicopathologic and Immunohistochemical Study of 9 Families. Am J Surg Pathol. 2012 Apr;36(4):589.

25. Griese M, Zarbock R, Costabel U, Hildebrandt J, Theegarten D, Albert M, et al. GATA2 deficiency in children and adults with severe pulmonary alveolar proteinosis and hematologic disorders. BMC Pulm Med. 2015 Aug 12;15(1):87.

26. Nalysnyk L, Rotella P, Simeone JC, Hamed A, Weinreb N. Gaucher disease epidemiology and natural history: a comprehensive review of the literature. Hematology. 2017 Feb 7;22(2):65–73.

27. Svensson CK, Feldt-Rasmussen U, Backer V. Fabry disease, respiratory symptoms, and airway limitation – a systematic review. Eur Clin Respir J [Internet]. 2015 Jan 1 [cited 2023 Oct 24]; Available from: https://www.tandfonline.com/doi/full/10.3402/ecrj.v2.26721

28. El-Chemaly S, Young LR. Hermansky-Pudlak Syndrome. Clin Chest Med. 2016 Sep 1;37(3):505–11.

29. Montani D, Coulet F, Girerd B, Eyries M, Bergot E, Mal H, et al. Pulmonary Hypertension in Patients With Neurofibromatosis Type I. Medicine (Baltimore). 2011 May;90(3):201.

30. Corut A, Senyigit A, Ugur SA, Altin S, Ozcelik U, Calisir H, et al. Mutations in SLC34A2 Cause Pulmonary Alveolar Microlithiasis and Are Possibly Associated with Testicular Microlithiasis. Am J Hum Genet. 2006 Oct 1;79(4):650–6.

31. Mauhin W, Habarou F, Gobin S, Servais A, Brassier A, Grisel C, et al. Update on Lysinuric Protein Intolerance, a Multi-faceted Disease Retrospective cohort analysis from birth to adulthood. Orphanet J Rare Dis. 2017 Jan 5;12(1):3.

32. Schuchman EH. The pathogenesis and treatment of acid sphingomyelinase-deficient Niemann–Pick disease. J Inherit Metab Dis. 2007;30(5):654.

33. Taveira-Dasilva AM, Moss J. Epidemiology, pathogenesis and diagnosis of lymphangioleiomyomatosis. Expert Opin Orphan Drugs. 2016 Apr 2;4(4):369–78.

34. Krenke K, Szczałuba K, Bielecka T, Rydzanicz M, Lange J, Koppolu A, et al. FARSA mutations mimic phenylalanyl-tRNA synthetase deficiency caused by FARSB defects. Clin Genet. 2019;96(5):468–72.

35. Antonellis A, Oprescu SN, Griffin LB, Heider A, Amalfitano A, Innis JW. Compound heterozygosity for loss-of-function FARSB variants in a patient with classic features of recessive aminoacyl-tRNA synthetase-related disease. Hum Mutat. 2018;39(6):834–40.

36. Has C, Spartà G, Kiritsi D, Weibel L, Moeller A, Vega-Warner V, et al. Integrin α3 Mutations with Kidney, Lung, and Skin Disease. N Engl J Med. 2012 Apr 19;366(16):1508–14.

37. Taub MA, Conomos MP, Keener R, Iyer KR, Weinstock JS, Yanek LR, et al. Genetic determinants of telomere length from 109,122 ancestrally diverse whole-genome sequences in TOPMed. Cell Genomics. 2022;2(1):100084.

38. Codd V, Nelson CP, Albrecht E, Mangino M, Deelen J, Buxton JL, et al. Identification of seven loci affecting mean telomere length and their association with disease. Nat Genet. 2013 Apr;45(4):422–7.

39. Dorajoo R, Chang X, Gurung RL, Li Z, Wang L, Wang R, et al. Loci for human leukocyte telomere length in the Singaporean Chinese population and trans-ethnic genetic studies. Nat Commun. 2019 Jun 6;10(1):2491.

40. Saxena R, Bjonnes A, Prescott J, Dib P, Natt P, Lane J, et al. Genome-Wide Association Study Identifies Variants in Casein Kinase II (CSNK2A2) to be Associated With Leukocyte Telomere Length in a Punjabi Sikh Diabetic Cohort. Circ Cardiovasc Genet. 2014 Jun;7(3):287–95.

41. Pooley KA, Bojesen SE, Weischer M, Nielsen SF, Thompson D, Amin Al Olama A, et al. A genome-wide association scan (GWAS) for mean telomere length within the COGS project: identified loci show little association with hormone-related cancer risk. Hum Mol Genet. 2013 Dec 15;22(24):5056–64.
